# Where Fluoride Is Present, Hexafluorosilicate Might Be Encountered: Supramolecular Binding of the SiF_6_^2–^ Anion by Nanojars

**DOI:** 10.1021/acsomega.4c08535

**Published:** 2024-10-18

**Authors:** Wisam A. Al Isawi, Matthias Zeller, Gellert Mezei

**Affiliations:** † Department of Chemistry, Western Michigan University, Kalamazoo, Michigan 49008, United States; ‡ Department of Chemistry, Purdue University, West Lafayette, Indiana 47907, United States

## Abstract

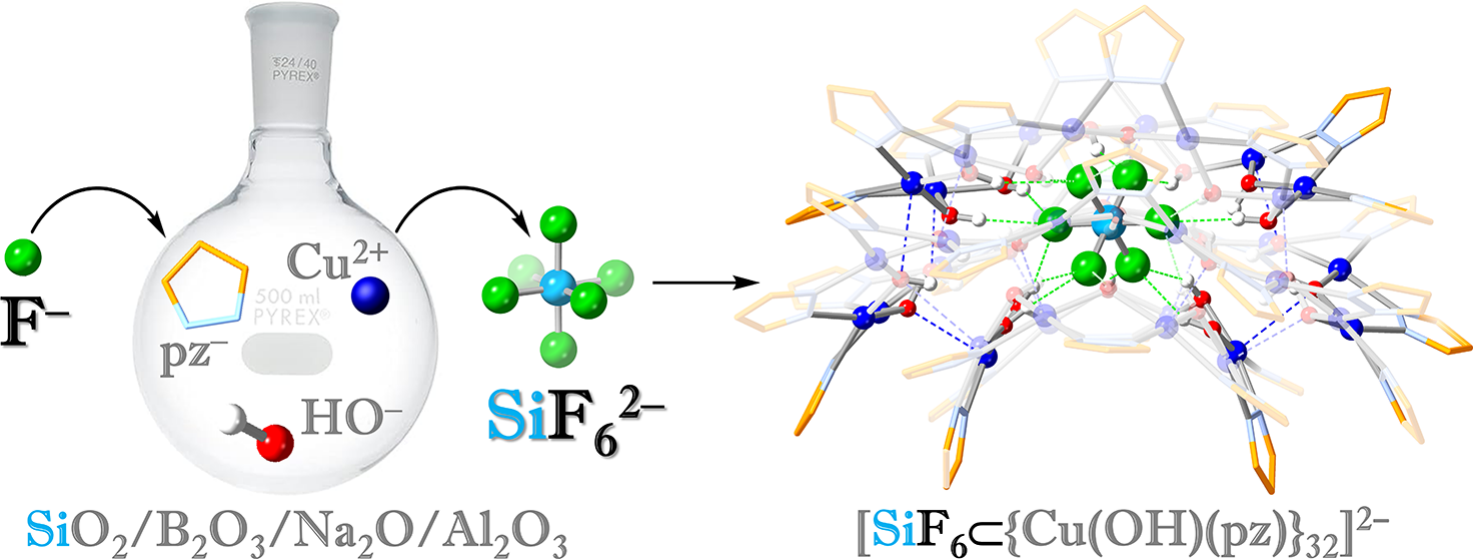

While the fact that HF etches glass is rather common chemical knowledge, the reaction of fluoride ions in an organic solvent with glassware under alkaline conditions has not yet been documented. It becomes apparent that, in general, whenever any fluorine-containing material is handled in silicon-based glassware, the possible presence of SiF_6_^2–^ should be considered regardless of the pH of the reaction medium. Subsequent to the initial, inadvertent synthesis by the reaction of CuF_2_ with laboratory glassware, hexafluorosilicate-entrapping supramolecular assemblies (nanojars) of the formula [SiF_6_⊂{*cis*-Cu^II^(μ–OH)(μ-pz)}*_n_*]^2–^ (**Cu***_**n**_***SiF**_**6**_; *n* = 28, 30, 32, 34) were rationally prepared by the reaction of Cu^2+^ with pyrazole (Hpz) in the presence of a base and SiF_6_^2–^ ions or by combining **Cu***_**n**_***CO**_**3**_ with H_2_SiF_6_ or HF. This work demonstrates that nanojars are excellent anion-binding agents not only for trigonal planar/pyramidal or tetrahedral anions but also for larger, octahedral anions, which are bound exclusively by charge-assisted hydrogen bonds. The SiF_6_^2–^ anion guest is preferentially bound by a larger Cu_32_ host, whereas CO_3_^2–^ favors a smaller Cu_27_ nanojar. The **Cu***_**n**_***SiF**_**6**_ mixture was characterized by electrospray ionization mass spectrometry as well as variable-temperature, paramagnetic ^1^H and ^19^F NMR spectroscopy in solution, complemented by single-crystal X-ray diffraction studies in the solid state, which reveal the unprecedented structure of a Cu_8+14+10_ nanojar (where Cu_8_, Cu_10_, and Cu_14_ are the component Cu_*x*_ metallamacrocycles of the Cu_32_ nanojar).

## Introduction

Hexafluorosilicate (SiF_6_^2–^) is a toxic inorganic anion that was thrust into the limelight after provoking concerns over its use in community water fluoridation.^1,2^ Amid continued controversy about the health benefits vs toxicity of fluoride,^3−6^ the Center for Disease Control and Prevention (CDC) recommends all public drinking water supplies contain 0.7 ppm of F^–^ to prevent tooth decay.^7,8^ Meanwhile, the U.S. Environmental Protection Agency (EPA) established a Maximum Contaminant Level for F^–^ of 4.0 ppm.^9^ Since the early 1950s, the most common water fluoridation agent in the U.S. is hexafluorosilicic acid (H_2_SiF_6_), which is obtained in large amounts as a byproduct of fertilizer production from phosphate rock (∼95%) and of the manufacture (from fluorspar) and use of HF in the solar panel and electronics industry for etching silicates and glasses (∼5%).^10^ Natural hexafluorosilicate minerals are rare; only a few examples are known, which are found as sublimates near volcanic fumaroles (as the Na^+^ and K^+^ salts) and/or burning coal seams (as the NH_4_^+^ salt).^11^ In the soil, hexafluorosilicate is thermodynamically unstable, where Al and Fe compete for complexing fluoride.^12^ Besides water fluoridation, H_2_SiF_6_ and its salts have a variety of applications including dentifrices, hardening cement and ceramics, as a precursor to synthetic cryolite (Na_3_AlF_6_) for the electrolytic production of aluminum, in the Betts electrolytic process for the purification of lead, as a wood preservative, as an additive in metal and stone floor finishing, and the manufacture of optical glass.^13^

Concern about the use of SiF_6_^2–^ as a water fluoridation agent began when the completeness of hexafluorosilicate hydrolysis to fluoride was questioned, and its interaction with lead pipelines leading to increased levels of Pb^2+^ in the blood was suggested.^14^ The Si–F bonds in SiF_6_^2–^ are considerably ionic: the Pauling electronegativity (EN) difference between Si (1.90) and F (3.98) is 2.08, with atomic charges on Si and F of +2.12 and −0.69.^15−18^ In comparison, other inorganic anions such as CO_3_^2–^ and SO_4_^2–^ are mostly covalent (with corresponding EN differences between C/S and O of 0.89 and 0.86, respectively). Yet, for a long time SiF_6_^2–^ was regarded as a stable species that resists hydrolysis.^19^ For example, a ∼ 1 M aqueous solution of (NH_4_)_2_SiF_6_ shows no detectable hydrolysis by ^19^F NMR spectroscopy.^20^ More recently, it has been demonstrated that the hydrolysis of SiF_6_^2–^ is concentration and pH dependent, and that at the concentration (<10^–5^ M SiF_6_^2–^) used for fluoridation of drinking water (pH 6.5–8.5), SiF_6_^2–^ is completely hydrolyzed.^21^

Yet, the SiF_6_^2–^ ion can easily form from silica/silicates and even silicon in the presence of fluoride, usually under acidic conditions where HF is expected to form.^22−25^ In fact, SiF_6_^2–^ is often encountered serendipitously when species containing labile fluorine atoms, such as the commonly used BF_4_^–^ and PF_6_^–^ anions,^26−31^ or less common species such as OTeF_5_^–^, come in contact with borosilicate glassware.^32^ Unexpected formation of SiF_6_^2–^ has even been observed from organofluorine compounds.^33^ It is likely that in many of the other structures in the Cambridge Structural Database (CSD)^34^ that contain SiF_6_^2–^ (765 entries in total), the hexafluorosilicate ion originates from glass. As an intentional counteranion for the crystallization of cationic compounds, SiF_6_^2–^ is much less commonly used compared to PF_6_^–^ (28,363 entries in the CSD) and BF_4_^–^ (19,953 entries in the CSD). SiF_6_^2–^ does not tend to form clusters, as opposed to other significantly ionic anions, such as AlF_6_^3–^ (EN difference: 2.37) and BeF_4_^2–^ (EN difference: 2.41), which form a variety of [Al_*x*_F_*y*_]^(3*x*−*y*)–^ and [Be_*x*_F_*y*_]^(2*x*−*y*)–^ clusters and polymers.^34^

Due to their environmental relevance, the supramolecular binding and extraction of various anions has been gaining increasing attention.^35−40^ Comparatively, the binding of the SiF_6_^2–^ ion is less extensively explored. Reported receptors include pure organic,^41,42^ as well as metal–organic cages, with^43−47^ or without coordination to the metal.^48−51^ SiF_6_^2–^ has also been bound into organic (not metal-coordinated)^52,53^ and metal–organic capsules (metal-coordinated),^54^ as well as organic^55^ and metal–organic macrocycles^56^ and an Ag_24_ cluster.^57^

**Scheme 1:**
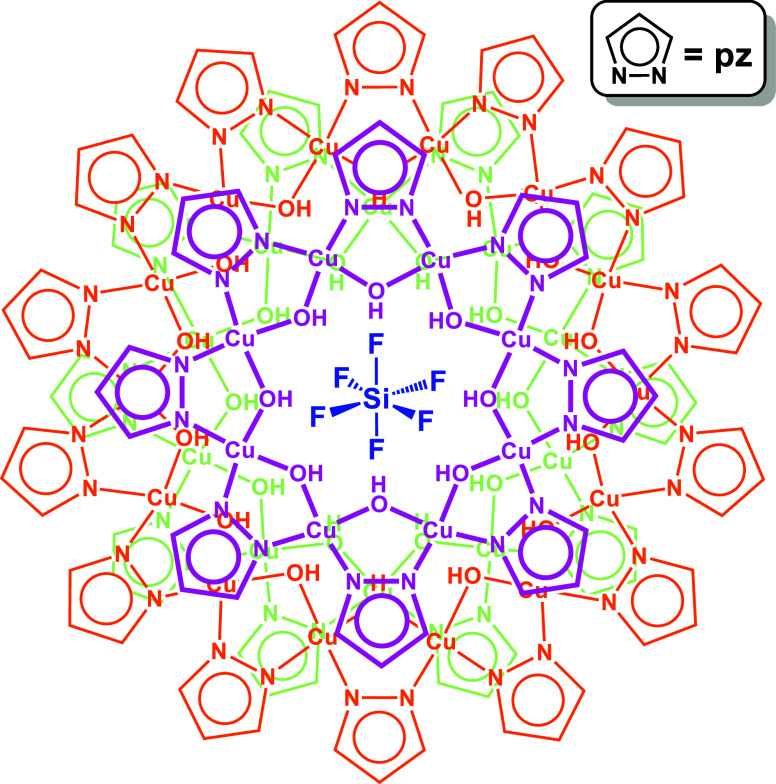
Schematic Representation of the [SiF_6_⊂{*cis*-Cu^II^(μ–OH)(μ-pz)}_8+14+10_]^2–^ Nanojar Color code: magenta, Cu_8_ ring; orange, Cu_14_ ring; green, Cu_10_ ring.

In this work, we report the binding of the SiF_6_^2–^ ion originating either in laboratory glassware or H_2_SiF_6_ within supramolecular containers termed *nanojars*, of the formula (Bu_4_N)_2_[SiF_6_⊂{*cis*-Cu^II^(μ–OH)(μ-pz)}*_n_*] (**Cu***_**n**_***SiF**_**6**_; *n* = 28, 30, 32, 34) (Scheme 1). Nanojars have emerged as a unique class of neutral anion-binding agents capable of sequestering a number of different tetrahedral or trigonal planar/pyramidal anions, including CO_3_^2–^, SO_4_^2–^ and RPO_3_^2–^ (R = H, OH, alkyl or aryl), as well as the highly toxic CrO_4_^2–^ and BeF_4_^2–^ ions.^58−65^ An intricate network of hydrogen bonds responsible for the strong binding of the hexafluorosilicate ion becomes evident from the single-crystal X-ray structure of the most abundant species (*n* = 32) in the nanojar mixture obtained by self-assembly or by anion exchange from carbonate nanojars (Bu_4_N)_2_[CO_3_⊂{*cis*-Cu^II^(μ–OH)(μ-pz)}*_n_*] (**Cu***_**n**_***CO**_**3**_; *n* = 27, 29–31). The SiF_6_-nanojars are characterized by electrospray ionization mass spectrometry (ESI-MS) and variable-temperature (VT), paramagnetic ^1^H and ^19^F nuclear magnetic resonance (NMR) spectroscopy in CH_3_CN and dimethyl sulfoxide-*d*_6_ (DMSO-*d*_6_) solutions, respectively. No noticeable solvents effects on the composition of nanojar mixtures were observed with the solutions in different solvents used for these studies.

## Results and Discussion

### Synthesis and Mass Spectrometry

**Figure 1:**
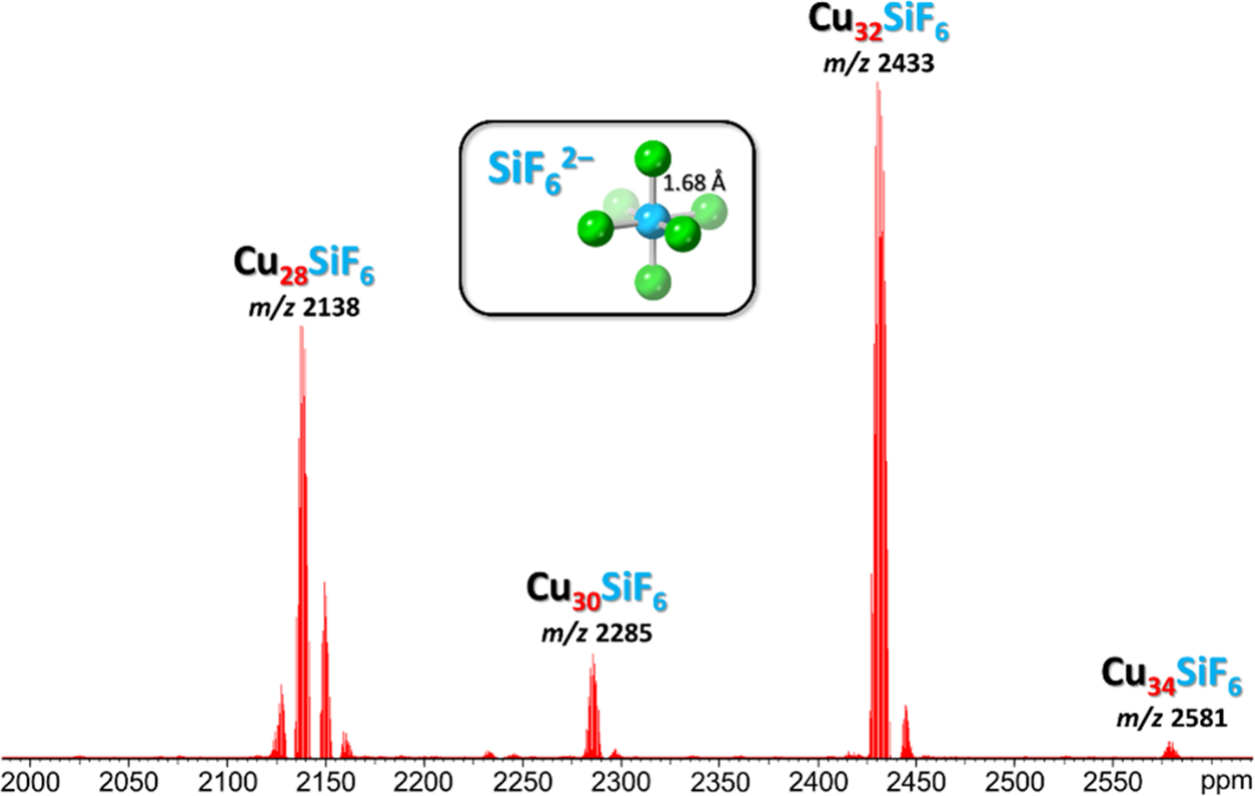
ESI-MS(−) spectrum in CH_3_CN of the as-synthesized hexafluorosilicate-entrapping nanojar mixture [SiF_6_⊂{Cu(OH)(pz)}*_n_*]^2–^ (**Cu***_**n**_***SiF**_**6**_; *n* = 28, 30, 32, 34). Detailed isotopic distributions are shown in Figure S2.

Hexafluorosilicate-entrapping nanojars were first obtained serendipitously from the ambient-temperature reaction of CuF_2_, pyrazole, NaOH and Bu_4_NOH (1:1:2.16:0.075 molar ratio) in tetrahydrofuran (THF). Amid the presence of excess base, fluoride ions reacted with silicate in the glass container walls and led to the formation of SiF_6_^2–^ ions which templated the formation of Cu*_n_*SiF_6_ nanojars (*n* = 28, 30–32, 34). Besides the **Cu***_**n**_***SiF**_**6**_ species, **Cu**_**31**_**CO**_**3**_ is also observed in the ESI-MS spectrum of the product mixture (Figure S1). When the same reaction was carried out in the presence of solid Na_2_CO_3_ or NaF (in equimolar amounts relative to CuF_2_), **Cu***_**n**_***CO**_**3**_ species (*n* = 27, 29, 31) are obtained almost exclusively, with only minor amounts of SiF_6_-containing nanojars. Similar results were obtained using CuF_2_, pyrazole and Bu_4_NOH in a 1:1:2 molar ratio. The reaction of Cu(NO_3_)_2_, pyrazole, NaOH, Bu_4_NOH and Na_2_SiF_6_ in a 1:1:1.93:0.066:1 molar ratio failed to produce nanojars and mostly low-nuclearity species, such as [Cu_3_O(pz)_2_(NO_3_)_3_]^−^ (*m*/*z* 526.79) and [Cu_3_O(pz)_3_(NO_3_)_2_]^−^ (*m*/*z* 531.86) are observed in the corresponding ESI-MS spectrum. However, when the hexafluorosilicate source was switched to the THF-soluble (Bu_4_N)_2_SiF_6_ (obtained *in situ* from Bu_4_NOH and H_2_SiF_6_), its reaction with Cu(NO_3_)_2_, pyrazole and Bu_4_NOH in a 1:1:1:2 molar ratio produced a carbonate-free mixture of **Cu***_**n**_***SiF**_**6**_ nanojars which can be isolated from solution as the Bu_4_N^+^ salts. ESI-MS(−) analysis of the product reveals that the as-synthesized mixture consists mostly of **Cu**_**32**_**SiF**_**6**_ (*m*/*z* 2433.02) and **Cu**_**28**_**SiF**_**6**_ (*m*/*z* 2137.77), with small amounts of **Cu**_**30**_**SiF**_**6**_ (*m*/*z* 2285.40) and traces of **Cu**_**34**_**SiF**_**6**_ (*m*/*z* 2580.65) (Figure 1). Interestingly, this self-assembly process yields only even-membered nanojars with SiF_6_^2–^, as opposed to other anions, such as BeF_4_^2–^ which forms both odd- and even-membered nanojars (*n* = 27–32) under similar reaction conditions.^58^

### Conversion of Cu*_n_*CO_3_ to Cu*_n_*SiF_6_ Nanojars by Anion Exchange

**Figure 2:**
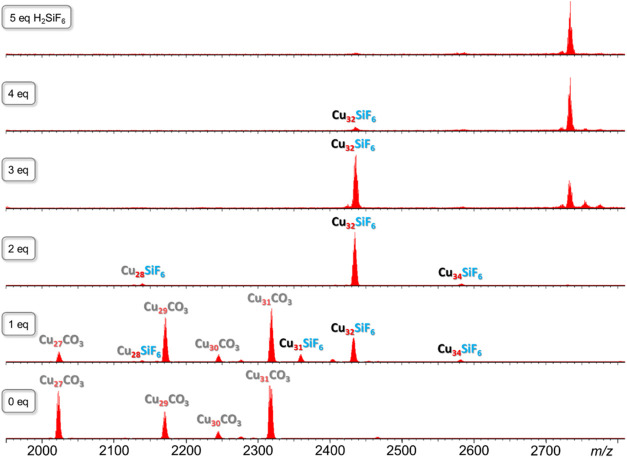
ESI-MS(−) spectra in CH_3_CN of **Cu***_**n**_***CO**_**3**_ (*n* = 27, 29–31) nanojars with varying amounts of added H_2_SiF_6_.

The titration of carbonate nanojars **Cu***_**n**_***CO**_**3**_ (*n* = 27, 29–31) with H_2_SiF_6_, monitored by ESI-MS(−), reveals that the addition of increasing amounts of the acid leads to a gradual and eventually complete transformation to hexafluorosilicate nanojars, **Cu***_**n**_***SiF**_**6**_ (*n* = 28, 32, 34), which subsequently decompose at higher acidities (Figure 2). Before adding any H_2_SiF_6_, the following **Cu***_**n**_***CO**_**3**_ species are observed: **Cu**_**27**_**CO**_**3**_ (*m*/*z* 2022.91), **Cu**_**29**_**CO**_**3**_ (*m*/*z* 2170.54), **Cu**_**30**_**CO**_**3**_ (*m*/*z* 2244.61) and **Cu**_**31**_**CO**_**3**_ (*m*/*z* 2318.16). At 1 equiv of H_2_SiF_6_, a drastic decrease in the amount of **Cu**_**27**_**CO**_**3**_ is noted, along with the appearance of **Cu***_**n**_***SiF**_**6**_ (*n* = 28, 31, 32, 34) species. Interestingly, **Cu**_**31**_**SiF**_**6**_ (*m*/*z* 2359.21) is present, which was absent in the as-synthesized mixture. Nevertheless, at 2 equiv of H_2_SiF_6_ this species disappears along with all **Cu***_**n**_***CO**_**3**_ nanojars, and only **Cu**_**32**_**SiF**_**6**_ remains with traces of **Cu**_**28**_**SiF**_**6**_ and **Cu**_**34**_**SiF**_**6**_. At 3 equiv of H_2_SiF_6_, only **Cu**_**32**_**SiF**_**6**_ is observed, and new, hitherto unidentified species (2– charged) appear in the *m*/*z* 2700–2800 region, together with low-nuclearity (Cu_3_) species (1– charged) in the *m*/*z* 440–600 region. Above 4 equiv of H_2_SiF_6_, **Cu**_**32**_**SiF**_**6**_ decomposes as well and the low-nuclearity species become dominant. The titration experiment shows that H_2_SiF_6_ first protonates the CO_3_^2–^ ion in **Cu***_**n**_***CO**_**3**_ to carbonic acid and replaces it with the SiF_6_^2–^ ion (**Cu***_**n**_***CO**_**3**_ + H_2_SiF_6_ → **Cu***_**n**_***SiF**_**6**_ + CO_2_ + H_2_O), while the nanojars rearrange to the most favorable size for the new guest (**Cu**_**32**_**SiF**_**6**_). Additional amounts of acid protonate the OH^–^ groups of the nanojar and break it down to low-nuclearity complexes, such as Cu_3_(OH)(pz)_3_X_3_ (where X is an anion or a solvent molecule).^59^

SiF_6_-nanojars can also be obtained form CO_3_-nanojars by titration with HF, which easily etches glass. As opposed to H_2_SiF_6_ which is a strong acid similar to H_2_SO_4_, with a second acid dissociation constant (p*K*_a2_) of 0.65 (earlier reported as 1.83),^66,67^ HF is a weak acid in solution (p*K*_a_ = 3.20).^68^ Consequently, a larger amount of HF is needed for the complete conversion of CO_3_- to SiF_6_-nanojars (Figure S3). At 1 equiv of added HF, only traces of **Cu***_**n**_***SiF**_**6**_ (*n* = 31, 32, 34) species are observed and the **Cu***_**n**_***CO**_**3**_ (*n* = 27, 29–31) composition appears unaffected. At 2 equiv of HF, the amount of **Cu**_**31**_**CO**_**3**_ is visibly reduced compared to the other **Cu***_**n**_***CO**_**3**_ species and the **Cu***_**n**_***SiF**_**6**_ species become more prominent. With increasing amounts of HF, the **Cu***_**n**_***CO**_**3**_ species gradually disappear and are replaced by **Cu***_**n**_***SiF**_**6**_. At 7 equiv of HF, the **Cu***_**n**_***CO**_**3**_ species are completely absent. Above 8 equiv of HF, **Cu**_**32**_**SiF**_**6**_ is the only species observed in the ESI-MS spectrum. Likely also a consequence of the relatively weak acidity of HF, no low-nuclearity copper complexes are observed even with a large excess of HF (30 equiv).

This titration experiment suggested a new method for the large-scale preparation of **Cu**_**32**_**SiF**_**6**_. Thus, treatment of an amount of **Cu***_**n**_***CO**_**3**_ (*n* = 27, 29–31) with 8 equiv of HF in acetonitrile solution, followed by the evaporation of the solvent, produces a pure sample of **Cu**_**32**_**SiF**_**6**_ (Figure S4).

Furthermore, treatment of **Cu***_**n**_***CO**_**3**_ (*n* = 27, 29–31) with HBF_4_ also leads to the formation of **Cu***_**n**_***SiF**_**6**_ (*n* = 32, 34). Similarly to HF, HBF_4_ etches glass to produce SiF_6_^2–^ ions. In this case, however, a clean transformation to SiF_6_-nanojars was not attained (Figure S5). With 2 equiv of HBF_4_, comparable amounts of **Cu**_**29**_**CO**_**3**_ and **Cu**_**32**_**SiF**_**6**_ are observed in the corresponding ESI-MS spectrum, whereas with 3 equiv of HBF_4_ only traces of nanojars are detected as they break down to low-nuclearity (mostly Cu_3_) species identified as Cu_3_(OH)(pz)_3_(HCOO) (*m*/*z* 453.88), Cu_3_(OH)(pz)_3_(BF_4_) (*m*/*z* 495.6), Cu_3_(OH)(pz)_3_(HCOO)_2_ (*m*/*z* 498.89) and Cu_3_(OH)(pz)_4_(HCOO) (*m*/*z* 520.95).

### Structural Analysis by X-ray Crystallography

**Figure 3:**
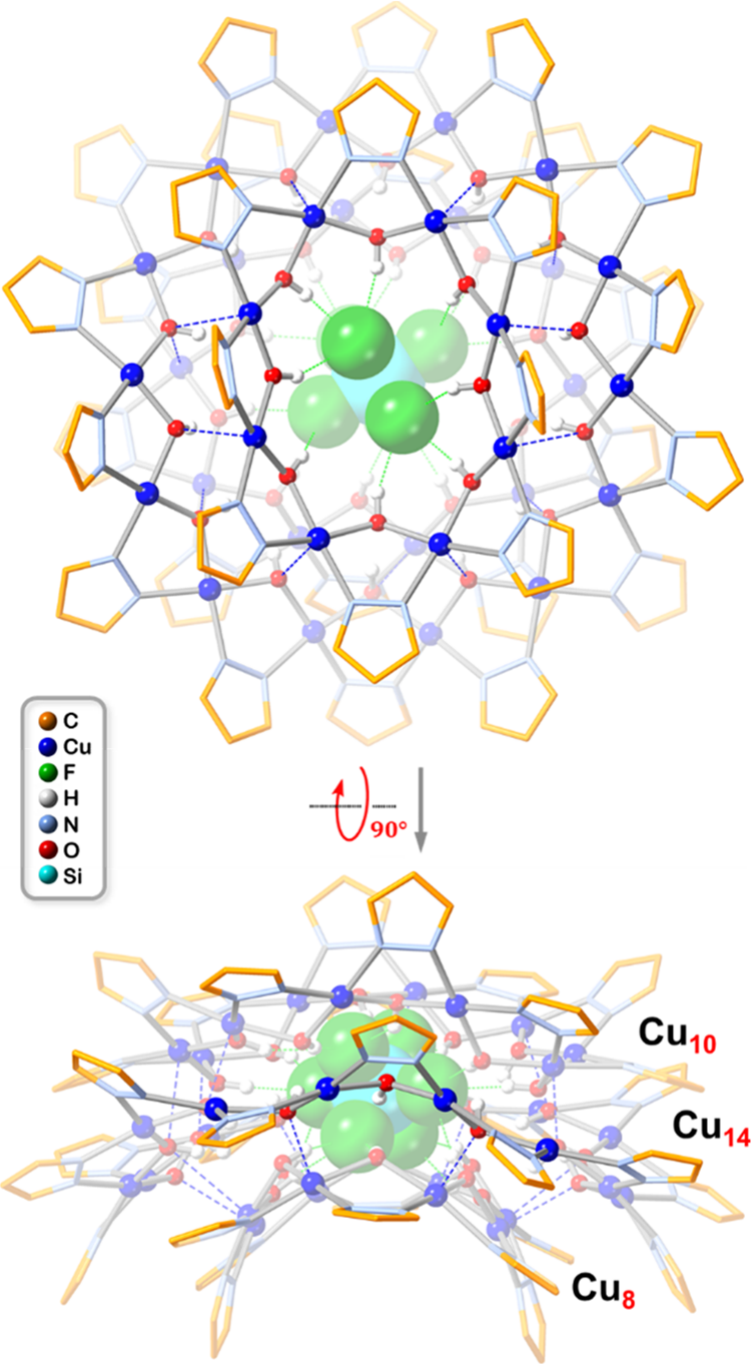
Ball-and-stick representation of the crystal structure of **1** (top- and side-views). Green and blue dotted lines indicate hydrogen bonds and axial Cu···O interactions, respectively. Counterions, lattice solvent molecules and C–H bond H atoms are omitted for clarity, and only the major SiF_6_^2–^ component is shown.

**Figure 4:**
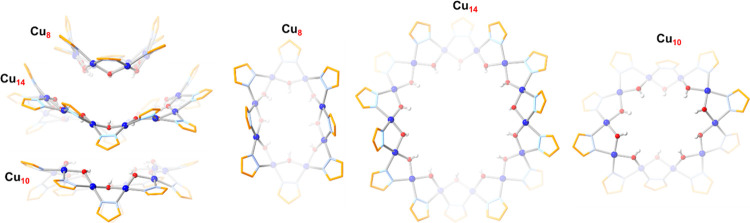
Deconstruction of the crystal structure of the **Cu**_**8+14+10**_**SiF**_**6**_ nanojar (**1**) showing the configuration of the individual Cu_8_, Cu_14_ and Cu_10_ rings (side- and top-views).

**Figure 5:**
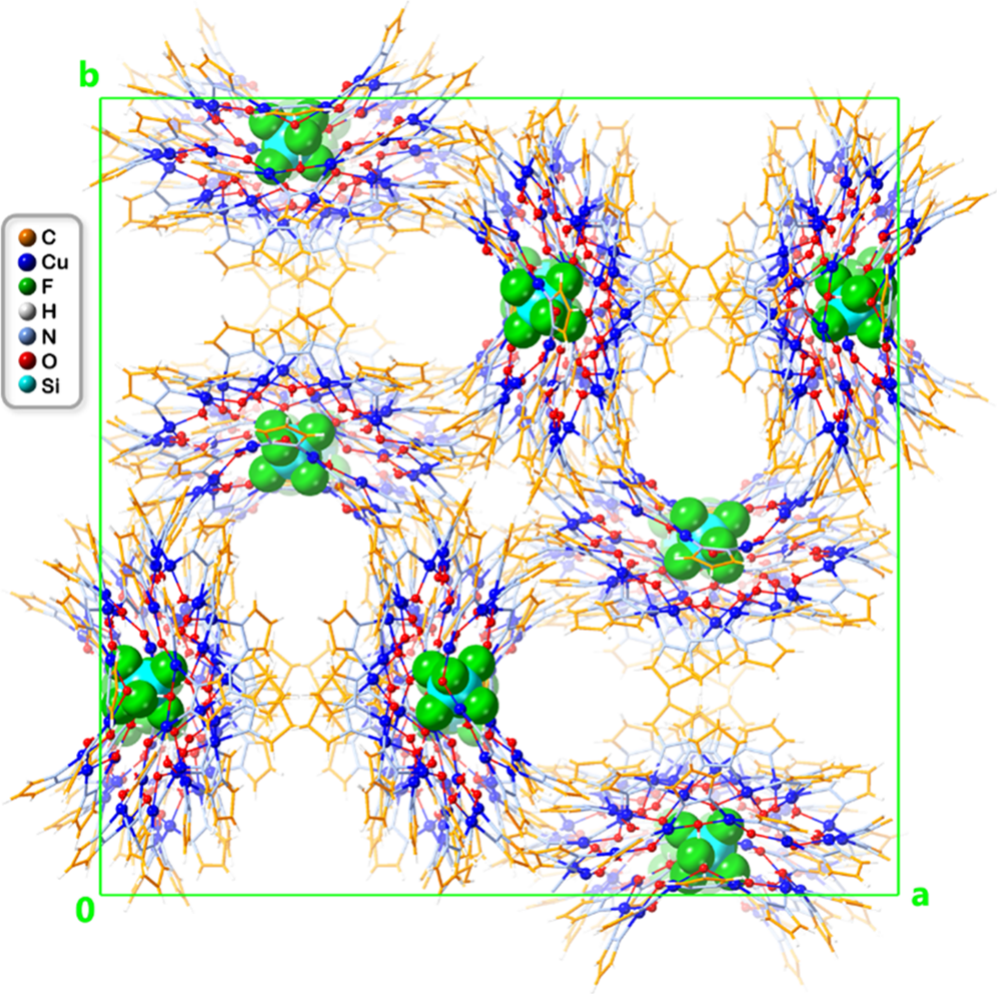
Packing diagram of **1** viewed along the *c* axis. Disordered counterions and lattice solvent molecules are omitted for clarity, and only the major SiF_6_^2–^ component is shown.

Single-crystal X-ray diffraction analysis of (Bu_4_N)_2_[SiF_6_⊂{*cis*-Cu^II^(μ–OH)(μ-pz)}_8+14+10_] (**Cu**_**32**_**SiF**_**6**_, **1**) provides unprecedented structural details of a nanojar composed of Cu_8_, Cu_14_ and Cu_10_ rings (Figures 3 and 4, Tables S1–S8). **1** crystallizes in the noncentrosymmetric tetragonal space group *I*4̅2*d* and is the first example of a nanojar crystal lattice with tetragonal symmetry. It is noteworthy that the space group *I*4̅2*d* (prototype: ice XII)^69^ is rather rare: only 813 (0.062%) out of a total of over 1.3 million structures in the CSD belong to this space group (2.8% of tetragonal structures, which represent 2.2% of all structures).^34^ The corresponding Schoenflies point group is *D*_*2d*_, with three *C*_2_ axes, an *S*_4_ axis and two σ_d_ mirror planes as symmetry elements. Within its crystal lattice, the nanojar units of **1** are located in general positions, surrounded by disordered Bu_4_N^+^ counterions and chlorobenzene and *n*-pentane solvent molecules (Figure 5).

The SiF_6_^2–^ ion centered in the cavity of the nanojar is found disordered in an 86/14 ratio. The observed Si–F bond lengths (Table S7) are similar to the average value of 1.68 Å obtained from the CSD.^34^ In the more favored, major orientation, the SiF_6_^2–^ ion accepts 18 O–H···F hydrogen bonds (with O···F distances shorter than 3.35 Å) from the Cu_8_ and Cu_10_ side rings (nine from each), ranging from 2.740(6) to 3.228(12) Å (average: 2.933(6) Å). In the minor orientation, only 16 such H bonds are formed with the Cu_8_ and Cu_10_ rings (eight with each), ranging from 2.68(2) to 3.34(3) Å (average: 3.04(3) Å). The Cu_14_ ring is too large to form H bonds with the SiF_6_^2–^ ion; instead, its OH groups are involved in H bonds with the Cu_8_ and Cu_10_ rings (described below). Thus, the nanojar cavity provides a snug fit for the SiF_6_^2–^ ion, which has a thermochemical radius of 2.48 Å and a corresponding molecular volume of 112 Å^3^.^70,71^

The nanojar scaffold of **1** consists of three Cu_*x*_ rings (*x* = 8, 14 and 10), bound together by axial Cu···O interactions and hydrogen bonds. Thus, there are eight and six Cu···O distances shorter than the sum of the van der Waals radii of Cu and O (2.92 Å) between O atoms of the central Cu_14_ ring and Cu atoms of the Cu_8_ and Cu_10_ rings, ranging from 2.342(4) to 2.900(5) Å (average: 2.538(5) Å) and from 2.358(4) to 2.833(5) Å (average: 2.548(5) Å), respectively. All Cu atoms of the Cu_14_ ring are four-coordinate, with no nearby axial O atoms. The four- or five-coordinate geometry of the Cu atoms in different Cu_*x*_ rings in **1** was analyzed using the coordination geometry indexes τ_4_ and τ_5_ (Table S6).^72,73^ In the case of τ_4_, a value of 1.00 indicates a perfect tetrahedral geometry and a value of zero corresponds to a perfect square planar geometry, whereas in the case of τ_5_, 1.00 and zero designate perfect trigonal bipyramidal and perfect square pyramidal geometries, respectively. The τ_4_ values in **1** range from 0.03 to 0.18 with an average of 0.11, whereas the τ_5_ values range from 0.00 to 0.19 with an average of 0.07, indicating that most of the Cu atoms have coordination geometries very close to square planar or square pyramidal.

Besides the O atoms that form axial Cu···O interactions, the central Cu_14_ ring also contributes 12 O–H···O hydrogen bonds (with donor–acceptor distances shorter than 3.20 Å) to the Cu_8_ and Cu_10_ side rings (six to each), with O···O distances ranging from 2.756(6) to 2.887(6) Å (average: 2.822(6) Å) and from 2.746(6) to 2.822(6) Å (average: 2.789(6) Å), respectively. The structural parameters within the Cu_*x*_-rings of **1**, such as average Cu–O and Cu–N bond lengths of 1.929(5) and 1.972(6) Å, average *trans* and *cis* N–Cu–O angles of 172.0(2) and 85.7(2)°, respectively, and average Cu···Cu distances of 3.300(2) Å (Table S2) are similar to those observed with other entrapped anions. Moreover, the descriptors of the Cu_*x*_ ring geometry defined earlier,^60,61^ namely the dihedral angles and the component fold and twist angles between the mean planes of adjacent pyrazolate moieties (average: 45.0(4), 41.2(4) and 20.9(4)°; Table S3) and between the mean planes of pyrazolate moieties and adjacent Cu–O–Cu units (average: 45.7(4), 4.3(4), and 45.1(4)°; Table S4) are also comparable to those observed in other nanojars.

Although it does bear some resemblance to the **Cu**_**32**_**BeF**_**4**_ nanojar (Cu_9+14+9_) described earlier (which crystallizes in the noncentrosymmetric, orthorhombic space group *P*2_1_2_1_2_1_),^58^ the overall shape of **1** (**Cu**_**8+14+10**_**SiF**_**6**_) shows significant differences. Specifically, the saddle-shape of the Cu_14_ ring is much less pronounced in **Cu**_**8+14+10**_**SiF**_**6**_ (largest deviation of a Cu atom from the Cu_14_ mean-plane: 1.543 Å; average deviation: 0.887 Å; Table S5) compared to Cu_9+14+9_BeF_4_ (with corresponding values of 1.977 and 1.194 Å, respectively). Likewise, the taco-shape of the Cu_8_ and Cu_10_ rings is less pronounced in **Cu**_**8+14+10**_**SiF**_**6**_ (largest deviation of a Cu atom from the Cu_8_ and Cu_10_ mean planes: 0.592 and 0.940 Å; average deviations: 0.460 and 0.644 Å) than in **Cu**_**9+14+9**_**BeF**_**4**_ (with corresponding values for the two Cu_9_ rings of 1.144/1.163 Å and 0.840/0.807 Å, respectively). The cavities of the taco-shaped Cu_8_ and Cu_10_ rings are filled by the Bu_4_N^+^ counterions.

### Variable-Temperature ^1^H NMR Spectroscopy

**Figure 6:**
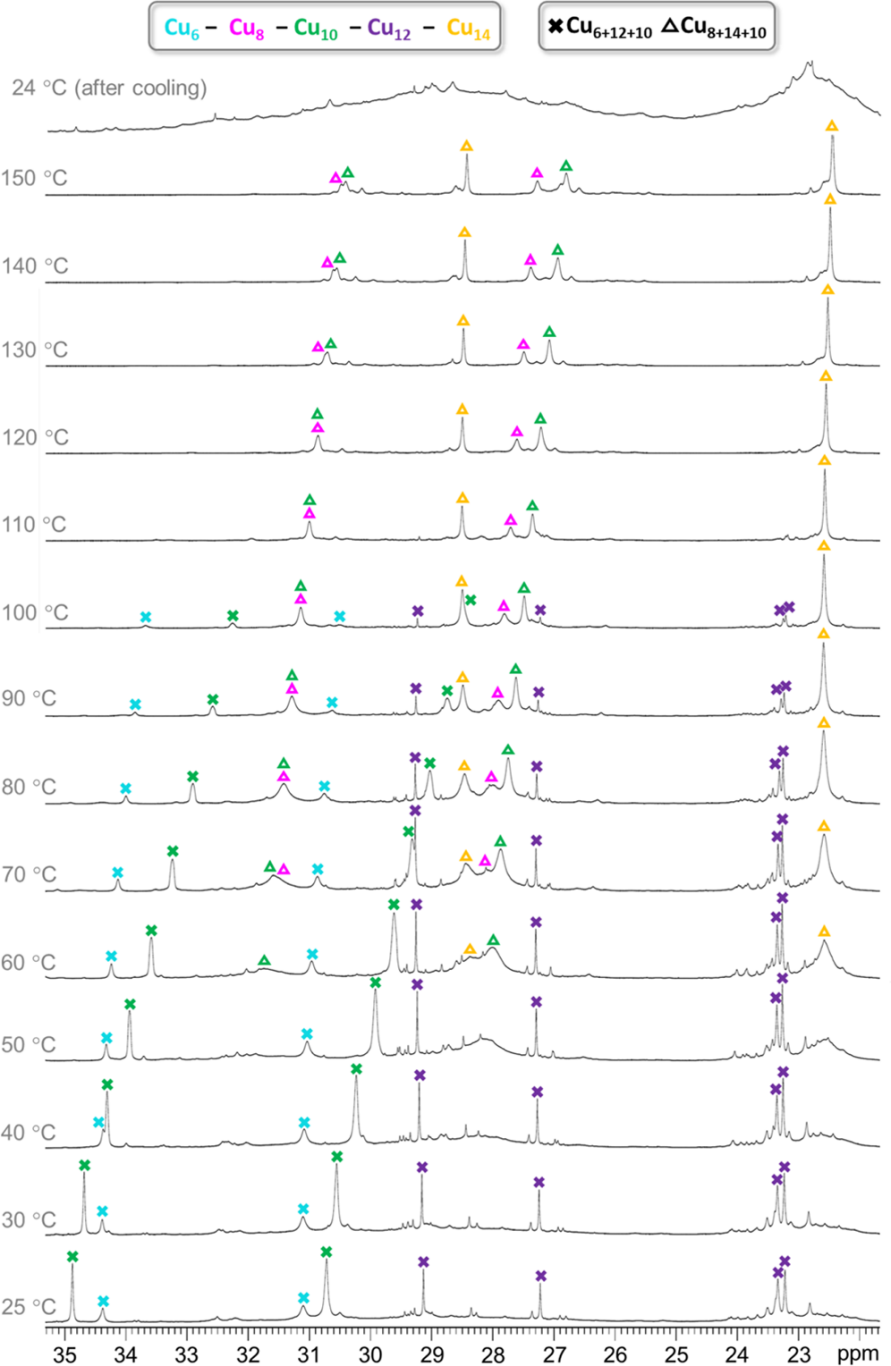
Variable-temperature ^1^H NMR spectra of the **Cu***_**n**_***SiF**_**6**_ (Cu*_n_*; *n* = 28, 30, 32, 34) nanojar mixture in DMSO-*d*_6_, showing pyrazolate proton signals in the 22–35 ppm window. Chemical shift values are given in Table S9. The temperatures shown are the target temperatures of the probe.

**Figure 7:**
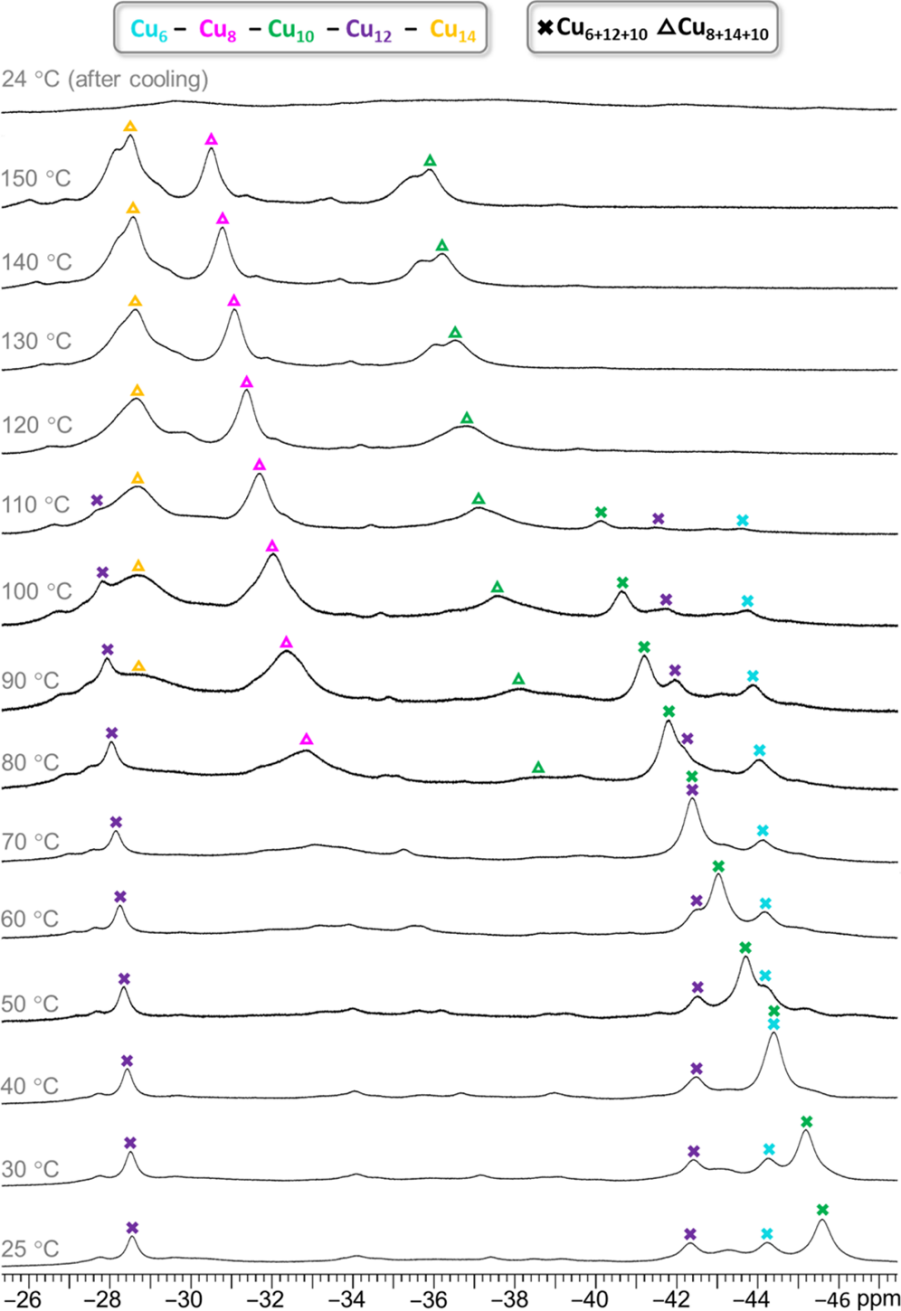
Variable-temperature ^1^H NMR spectra of the **Cu***_**n**_***SiF**_**6**_ (Cu*_n_*; *n* = 28, 30, 32, 34) nanojar mixture in DMSO-*d*_6_, showing OH proton signals in the −26 to −47 ppm window. Chemical shift values are given in Table S9. The temperatures shown are the target temperatures of the probe.

As indicated by ESI-MS analysis (Figure 1), the as-synthesized **Cu***_**n**_***SiF**_**6**_ nanojar mixture contains comparable amounts of **Cu**_**28**_**SiF**_**6**_ and **Cu**_**32**_**SiF**_**6**_, and only minor amounts of **Cu**_**30**_**SiF**_**6**_ and **Cu**_**34**_**SiF**_**6**_. Yet, the ambient-temperature ^1^H NMR spectrum of **Cu***_**n**_***SiF**_**6**_ displays only one major set of signals, corresponding to **Cu**_**28**_**SiF**_**6**_ (Figures 6 and 7, Table S9). A VT-NMR experiment (25 to 150 °C in DMSO-*d*_6_) reveals that the apparent absence of the **Cu**_**32**_**SiF**_**6**_ signals at 25 °C is attributable to their extreme broadness at that temperature. On heating, however, the signals of the Cu_8_, Cu_14_ and Cu_10_ rings of **Cu**_**32**_**SiF**_**6**_ become increasingly sharper and are clearly distinguishable above 60 °C. Moreover, a gradual diminishing of the **Cu**_**28**_**SiF**_**6**_ signals is observed on heating, which virtually disappear above 110 °C. Since no new signals are observed in the NMR spectrum nor any changes in the appearance of the solution after the VT-NMR experiment, it is concluded that **Cu**_**28**_**SiF**_**6**_ completely transforms into **Cu**_**32**_**SiF**_**6**_ on heating to 150 °C. Indeed, after cooling to 25 °C following the VT-NMR experiment, only two extremely broad signals in the pz proton region (22–35 ppm window) centered at 23 and 29 ppm, corresponding to the Cu_14_ and the Cu_8_/Cu_10_ rings of **Cu**_**32**_**SiF**_**6**_, and an almost flat line in the OH proton region (−26 to −47 ppm window) are observed. As discussed below, the ^19^F NMR spectrum of the same sample confirms the survival of the **Cu**_**32**_**SiF**_**6**_ nanojar after heating to 150 °C in DMSO-*d*_6_, along with the decomposition of the **Cu**_**28**_**SiF**_**6**_ nanojar.

The conversion of **Cu**_**28**_**SiF**_**6**_ to **Cu**_**32**_**SiF**_**6**_ in hot DMSO-*d*_6_ likely occurs by a different mechanism than the conversion of **Cu***_**n**_***CO**_**3**_ to **Cu***_**n**_***SiF**_**6**_ in the presence of H_2_SiF_6_. In the former case, two of the Cu_*x*_ rings of **Cu**_**6+12+10**_**SiF**_**6**_ need to expand by two Cu(pz)(OH) units each to form **Cu**_**8+14+10**_**SiF**_**6**_, on the expense of another **Cu**_**28**_**SiF**_**6**_ nanojar. In contrast, the latter case involves not only changes in Cu_*x*_ ring sizes (from Cu_6+12+9_, Cu_7+13+9_, Cu_8+13+8_ and Cu_8+14+9_ to Cu_8+14+10_) but also anion exchange from CO_3_^2–^ to SiF_6_^2–^, and protonated species are possible due to the strong acidity of H_2_SiF_6_. No intermediates of either of these transformations were apparent in the NMR or ESI-MS spectra of the corresponding nanojar mixtures, suggesting that they are short-lived and quickly rearrange to the stable nanojar species observed.

**Figure 8:**
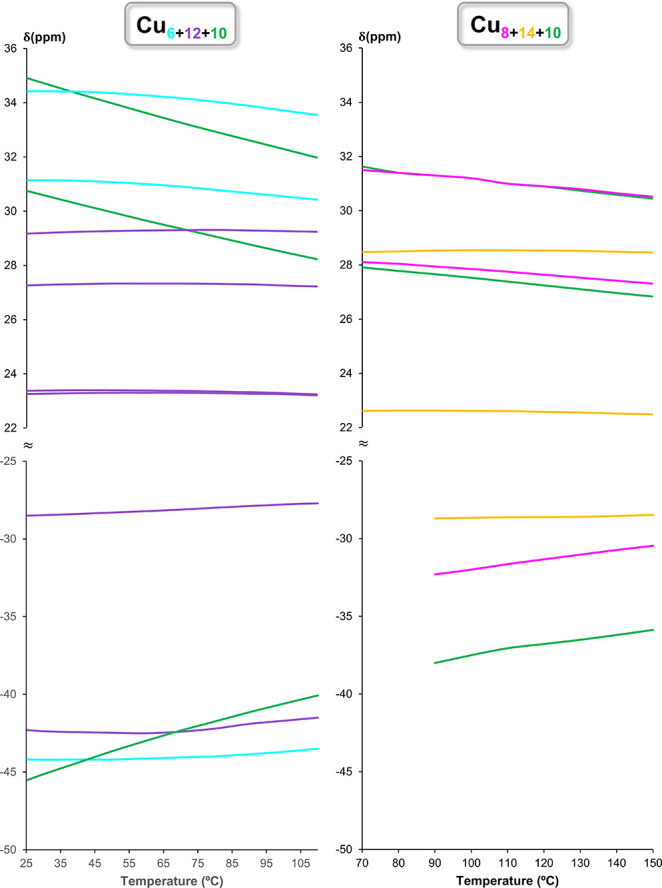
Temperature-dependent variation of the chemical shifts (δ) of different Cu_*x*_-ring protons in the **Cu**_**28**_**SiF**_**6**_ and **Cu**_**32**_**SiF**_**6**_ nanojars in DMSO-*d*_6_.

In contrast to its solid-state structure, which lacks symmetry, ^1^H NMR indicates that in solution all pz moieties as well as OH groups are identical within each Cu_*x*_ ring of **Cu**_**32**_**SiF**_**6**_. For each pz unit, two signals are observed in a 1:2 ratio, corresponding to the 4- and 3/5-positions. The broadness, along with the drastic downfield shift of the pz proton peaks and an even more drastic upfield shift of the OH proton peaks, as well as the loss of *J* coupling between nuclei is due to the paramagnetic Cu^2+^ centers. The VT-NMR experiment suggests a strengthening of the antiferromagnetic interactions between Cu centers at higher temperatures, which leads to a reduction of spin density on the individual Cu_*x*_ rings and consequently, to sharper ^1^H NMR signals. Of the three Cu_*x*_ rings of **Cu**_**32**_**SiF**_**6**_, Cu_10_ is the most paramagnetically shifted, followed by Cu_8_ and then by Cu_14_ at room temperature. At increasing temperatures, the corresponding peaks are less paramagnetically shifted, indicating a Curie behavior (magnetization inversely proportional to the temperature) (Figure 8). The temperature-dependence of the chemical shift is also the largest for the Cu_10_ ring, and smallest for the Cu_14_ ring.

Similarly to **Cu**_**32**_**SiF**_**6**_, all pz moieties as well as OH groups are identical within the Cu_*x*_ rings of **Cu**_**28**_**SiF**_**6**_, with the exception of the Cu_12_ ring, for which two sets of signals of approximately equal integration are observed both for the pz and the OH groups. The latter observation suggests that six pz units of the Cu_12_ ring experience a significantly different spin density than the other six pz units. Indeed, the crystal structures of the Cu_6+12+10_ nanojar (with BeF_4_^2–^, SO_4_^2–^, and CrO_4_^2–^ anions)^58,62,63^ indicate an alternating position of the pz units of the Cu_12_ ring, with half of them pointing toward the Cu_6_ ring and the other half pointing toward the Cu_10_ ring. The relative sharpness of the peaks of **Cu**_**28**_**SiF**_**6**_, even at ambient temperature, indicates a more efficient electronic communication and stronger antiferromagnetic interactions between its Cu centers. It also becomes apparent that the extent of the spin density transfer and the antiferromagnetic interaction between its Cu_10_ ring and the adjacent Cu_12_ ring is different from the one between the Cu_10_ and Cu_14_ rings of **Cu**_**32**_**SiF**_**6**_, as the chemical shifts of the Cu_10_ ring protons and their temperature-dependence in the two nanojars are quite different. For example, at 70 °C the ^1^H NMR resonances of the Cu_10_ ring pz protons appear at 29.36 and 33.27 ppm in **Cu**_**28**_**SiF**_**6**_, whereas in **Cu**_**32**_**SiF**_**6**_ the corresponding signals are found at 27.91 and 31.63 ppm. As in **Cu**_**32**_**SiF**_**6**_, the Cu_10_ ring of **Cu**_**28**_**SiF**_**6**_ appears to possess the largest spin density, indicated by the most prominent hyperfine shifts and the largest temperature-dependence of the corresponding signals. The corresponding Curie plots of the ^1^H NMR data, obtained by plotting δ as a function of 10^3^/T, are shown in Figure S7.

### Variable-Temperature ^19^F NMR Spectroscopy

**Figure 9:**
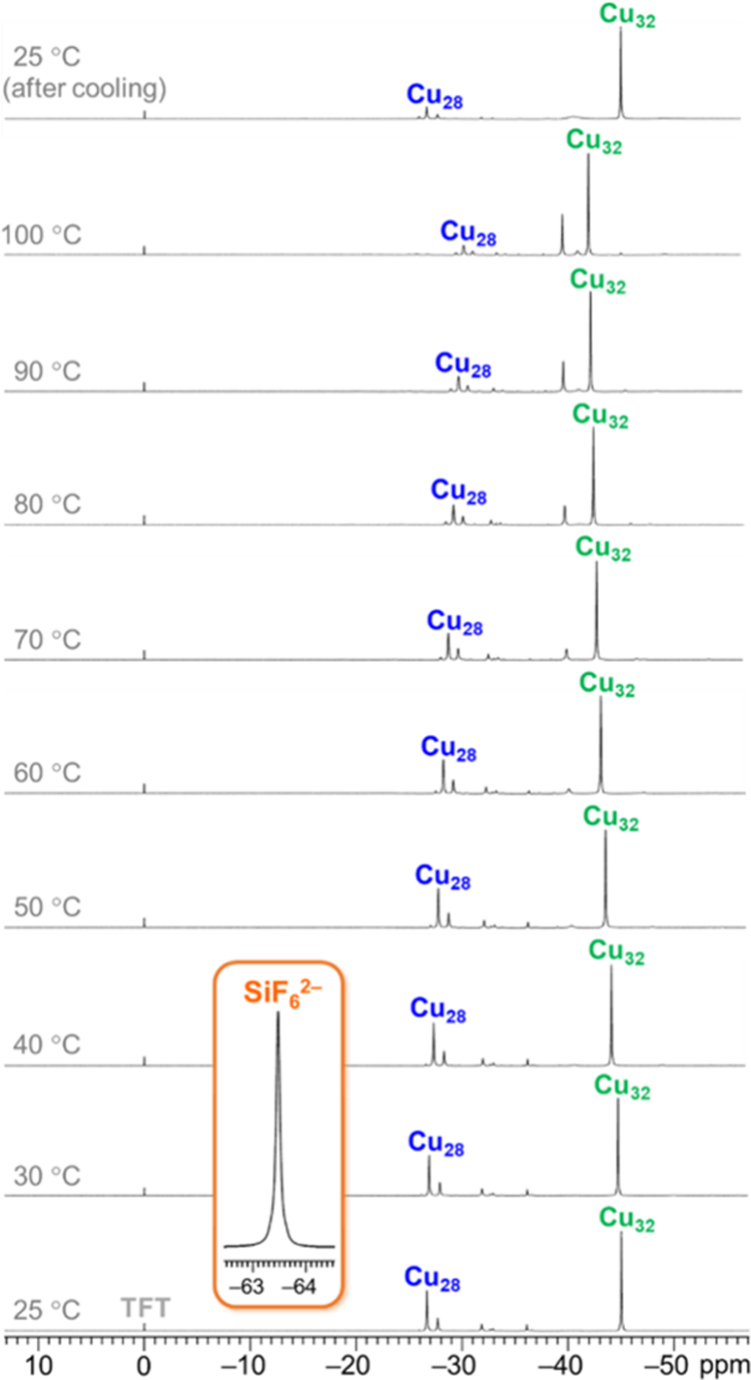
VT ^19^F NMR spectra of the **Cu***_**n**_***SiF**_**6**_ (Cu*_n_*; *n* = 28, 30, 32, 34) nanojar mixture in DMSO-*d*_6_, referenced to C_6_H_5_CF_3_ (TFT) as internal standard. Inset shows the signal of the free SiF_6_^2–^ ion as (Bu_4_N)_2_SiF_6_ in DMSO-*d*_6_ at 25 °C. Assignments were made based on correlations with ESI-MS and ^1^H NMR spectra, and the corresponding chemical shift values are given in Table S10.

**Figure 10:**
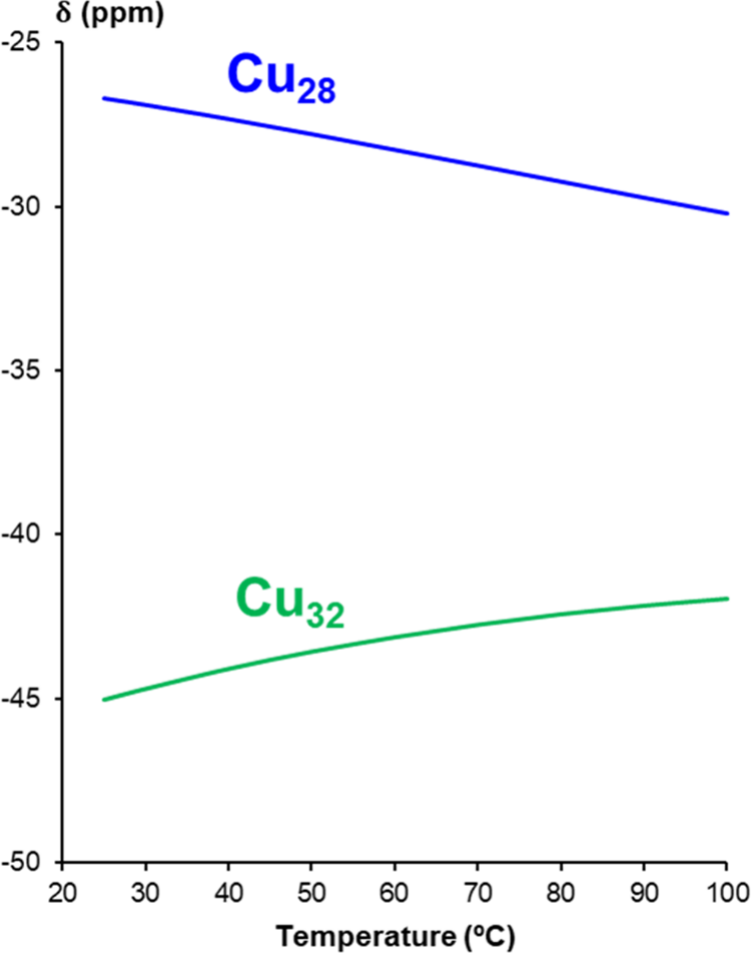
Temperature-dependent variation of the chemical shifts (δ) of the ^19^F atoms in the **Cu**_**28**_**SiF**_**6**_ and **Cu**_**32**_**SiF**_**6**_ nanojars in DMSO-*d*_6_.

Owing to the 100% natural abundance and high sensitivity of its ^19^F nuclei, the SiF_6_^2–^ anion can conveniently be probed directly by ^19^F NMR spectroscopy. The ^19^F NMR signal of the free SiF_6_^2–^ anion in (Bu_4_N)_2_SiF_6_ appears at −63.47 ppm at 25 °C in DMSO-*d*_6_ (referenced to C_6_H_5_CF_3_ as internal standard). Upon entrapment into nanojars, the ^19^F chemical shift of the SiF_6_^2–^ anion moves drastically downfield to −45.03 ppm in **Cu**_**32**_**SiF**_**6**_ and to −26.68 ppm in **Cu**_**28**_**SiF**_**6**_. The observation of a single signal for the six F atoms of SiF_6_^2–^ indicates that in contrast with the solid state structure, the position of the anion within the nanojar cavity is dynamic in solution. VT ^19^F NMR experiments display a temperature-dependence of the chemical shifts similar to the ^1^H NMR signals (Figure 9 and Table S10). However, only the signal of the **Cu**_**28**_**SiF**_**6**_ nanojar displays a Curie-type behavior (δ ∝ 1/*T*), whereas in the case of the **Cu**_**32**_**SiF**_**6**_ nanojar an anti-Curie behavior (δ ∝ *T*) is observed (Figures 10 and S8).

Because the ^1^H NMR signals of the **Cu**_**32**_**SiF**_**6**_ nanojar are excessively broad at ambient temperature, the composition of the sample after the VT ^1^H NMR experiment (heating to 150 °C in DMSO-*d*_6_) was confirmed by ^19^F NMR spectroscopy. Indeed, the ^19^F NMR spectrum of the sample shows that after cooling to 25 °C only the **Cu**_**32**_**SiF**_**6**_ nanojar is still present, whereas the **Cu**_**28**_**SiF**_**6**_ nanojar vanished (Figure S9).

## Conclusions

The previously unidentified ESI-MS peaks of the product of the reaction of CuF_2_ with pyrazole in the presence of excess base were recognized as belonging to **Cu***_**n**_***SiF**_**6**_ nanojars after SiF_6_^2–^ was deliberately used for nanojar formation. While the SiF_6_^2–^ ion is easily identifiable in an X-ray crystal structure, it is not immediately evident in NMR or mass spectra when its presence is not anticipated. Following their initial, serendipitous discovery, hexafluorosilicate-binding nanojars have been rationally synthesized and characterized by ESI-MS as well as variable temperature, paramagnetic ^1^H and ^19^F NMR spectroscopy in solution, and by single-crystal X-ray diffraction in the solid-state.

**Cu**_**32**_**SiF**_**6**_ and **Cu**_**28**_**SiF**_**6**_ were identified by ESI-MS as major species in the as-synthesized **Cu***_**n**_***SiF**_**6**_ nanojar mixture, with small amounts of **Cu**_**30**_**SiF**_**6**_ and **Cu**_**34**_**SiF**_**6**_ and occasionally **Cu**_**31**_**SiF**_**6**_. Thus, we have demonstrated that nanojars are capable of binding not only tetrahedral or trigonal planar/pyramidal anions, but also larger, octahedral anions. For the first time, the crystal structure of a Cu_8+14+10_ nanojar is described, detailing the intricate bonding pattern of the supramolecular binding of SiF_6_^2–^ exclusively by hydrogen bonds in **Cu**_**32**_**SiF**_**6**._ In contrast to only 10–15 hydrogen bonds to three or four O or F atoms in the case of trigonal planar/pyramidal or tetrahedral anions studied before, the six F atoms of the SiF_6_^2–^ ion participate in 18 hydrogen bonds with the nanojar cavity. Its greater electronegativity and lower polarizability makes F a poorer hydrogen bond acceptor than O^74−76^ and even Cl.^77^ Indeed, we observed that sulfate (SO_4_^2–^) is a better hydrogen bond acceptor than tetrafluoroberyllate (BeF_4_^2–^) in nanojars.^61^ Previous anion-binding studies also established an anti-Hofmeister selectivity for nanojars, with preferential binding of anions with the largest hydration energies, such as CO_3_^2–^ (Δ*G*_h_° = −1324 kJ/mol).^78^ Thus, interference from adventitious CO_3_^2–^ ions (found in trace amounts in reagents, especially bases) was encountered in the case of anions with smaller hydration energies, such as phosphonates (RPO_3_^2–^).^60^ Although the hydration energy of SiF_6_^2–^ is also considerably smaller (Δ*G*_h_° = −938 kJ/mol),^79^ no interference was observed from carbonate. In fact, carbonate nanojars (**Cu***_**n**_***CO**_**3**_) can be transformed into **Cu***_**n**_***SiF**_**6**_ by reaction with H_2_SiF_6_ and even with HF or HBF_4_, which etch the glass reaction container and produce SiF_6_^2–^ ions.

^1^H and ^19^F VT-NMR studies not only offer structural details of the differently sized nanojars in the as-synthesized **Cu***_**n**_***SiF**_**6**_ nanojar mixture, which are not evident from the ESI-MS measurements, but also shed light on their different thermal stability and reveal a different influence of their paramagnetic Cu^2+^ centers on the corresponding ^1^H and ^19^F hyperfine shifts. While only the Cu_6+12+10_ ring combination is expected for **Cu**_**28**_**SiF**_**6**_ based on previous observations, the ^1^H NMR experiments reveal that the **Cu**_**32**_**SiF**_**6**_ nanojar consists mostly of a Cu_8+14+10_ ring combination instead of the possible alternative, Cu_9+14+9_. VT-NMR studies confirm that the **Cu**_**32**_**SiF**_**6**_ nanojar is stable at 150 °C in a DMSO-*d*_6_ solution, whereas the **Cu**_**28**_**SiF**_**6**_ nanojar gradually decomposes on heating. The observation of a single set of signals for each Cu_*x*_ ring indicates that all pz units within each ring are equivalent, with the exception of the Cu_12_ ring in **Cu**_**6+12+10**_**SiF**_**6**_, which displays two different sets of signals corresponding to pz units neighboring either the Cu_6_ or the Cu_10_ ring. This high symmetry solution structure is possible only if the nanojar scaffold is fluxional, since no symmetry is observed in the corresponding crystal structure. The dynamic nature of the nanojar cavity translates to the entrapped anion as well, corroborated by the observation of a single ^19^F NMR signal for the six F atoms of the SiF_6_^2–^ ion. The VT-NMR studies indicate a temperature-dependence of the signals which follow Curie’s Law in the case of the ^1^H NMR signals, with the strongest temperature-dependence observed for the Cu_10_ ring of the Cu_6+12+10_ nanojar, and the Cu_8_ and Cu_10_ rings of the Cu_8+14+10_ nanojar. For the ^19^F NMR signals, a Curie-type behavior is only observed in the case of **Cu**_**28**_**SiF**_**6**_, whereas **Cu**_**32**_**SiF**_**6**_ displays an anti-Curie behavior. Surprisingly, even broader ^1^H NMR signals are observed for the Cu_8+14+10_ nanojar than for Cu_8+14+9_ (previously studied with other anions), despite the fact that in the all-even-membered rings of the former the antiparallel spins on consecutive Cu centers are all compensated (Cu···Cu distances: 3.126(1)–3.388(1) Å), whereas in the odd-membered Cu_9_ ring one of the spins remains uncompensated and is magnetically frustrated.^64^

Larger anions template the formation of larger nanojars. With the small CO_3_^2–^ anion (C–O: 1.28 Å), the preferred nanojar size is Cu_27_ (Cu_6+12+9_), whereas the larger SO_4_^2–^ anion (S–O: 1.48 Å) favors the larger Cu_31_ (Cu_8+14+9_) nanojar. With the even larger SiF_6_^2–^ anion (Si–F: 1.68 Å), the preferred nanojar size increases to Cu_32_ (Cu_8+14+10_). Furthermore, an even larger Cu_34_ nanojar has been detected for the first time in small amounts with SiF_6_^2–^, which features unprecedented, yet hitherto unidentified Cu_10+14+10_ or Cu_8+16+10_ ring combinations. To access this new nanojar in larger amounts for further characterizations, future studies will be conducted using even larger anions, such as GeF_6_^2–^ (Ge–F: 1.79 Å) and SnF_6_^2–^ (Sn–F: 1.95 Å). In addition to the Cu_34_ nanojar being expected to be more prevalent with them, these larger anions will also pave the way to conceivably even larger nanojars.

## Experimental Section

**WARNING!***Hydrofluoric acid (HF), hexafluorosilicic acid (H*_2_*SiF*_6_*) and tetrafluoroboric acid (HBF*_4_*) are highly corrosive and toxic. Both the fumes and very short contact with the liquid can cause severe and painful burns. Appropriate precautions should be taken while handling them*.

### General

All commercially available chemicals were used as received (solvents are ACS or HPLC grade, and THF is inhibited with 250 ppm BHT). Cu(NO_3_)_2_·2.5H_2_O (ACS reagent, 98%), CuF_2_·*x*H_2_O (*x* ≈ 2, 98%), NaOH (ACS reagent, 97%), HF (puriss. p.a., 40% in H_2_O), H_2_SiF_6_ (purum, 34% in H_2_O) and HBF_4_ (48% in H_2_O) were purchased from Sigma-Aldrich, pyrazole (99%) from Oakwood Chemical, and ^*n*^Bu_4_NOH (HPLC grade, 1.0 M in H_2_O) from Thermo Scientific. (Bu_4_N)_2_[CO_3_⊂{Cu(OH)(pz)}*_n_*] (Cu*_n_*CO_3_; *n* = 27, 29–31) was prepared according to the published procedure.^65^ Reported yields for nanojars are based on the Cu(II) starting materials. Deionized water was freshly boiled and cooled to room temperature under *N*_2_(*g*). NMR spectra were collected on a Jeol JNM-ECZS (400 MHz) instrument, using C_6_H_5_CF_3_ (TFT) as an internal standard (δ = 0.00 ppm) for the ^19^F spectra (376 MHz).

### Synthesis of (Bu_4_N)_2_[SiF_6_⊂{Cu(OH)(pz)}_32_] (Cu_32_SiF_6_) Using HF

A solution of (Bu_4_N)_2_[CO_3_⊂{Cu(OH)(pz)}*_n_*] (**Cu***_**n**_***CO**_**3**_; *n* = 27, 29–31) (0.1000 g, 0.0207 mmol) in acetonitrile (20 mL) was mixed with a 0.1 M solution of HF in acetonitrile (prepared from a 40% aqueous HF solution; 1.658 mL, 0.1658 mmol) in a borosilicate glass volumetric flask. After standing for 12 h in the stoppered flask, the solvent was evaporated to yield a dark-blue solid (0.0914 g), which was identified by ESI-MS to be pure **Cu**_**32**_**SiF**_**6**_ (Figure S4).

### Synthesis of (Bu_4_N)_2_[SiF_6_⊂{Cu(OH)(pz)}*_n_*] (Cu*_n_*SiF_6_; *n* = 28, 30, 32, 34) Using (Bu_4_N)_2_SiF_6_

In a 50 mL three-neck round-bottom flask equipped with a pressure-equalizing addition funnel and a stir bar, a solution of Cu(NO_3_)_2_·2.5H_2_O (1.0000 g, 4.30 mmol) and pyrazole (0.2927 g, 4.30 mmol) was prepared in THF (25 mL). H_2_SiF_6_ (34.2% in H_2_O, 1.8249 g, 4.30 mmol) was added to the reaction solution and stirred. Then, the reaction flask was purged with *N*_2_(*g*), and ^*n*^Bu_4_NOH (1 M in H_2_O, 17.20 mL, 17.20 mmol) was added dropwise via the addition funnel to the reaction mixture under stirring. The deep-blue solution was cannulated into water (500 mL) under stirring. The blue precipitate was filtered out and washed thoroughly with water. Drying in a high vacuum afforded a blue powder. Yield: 0.5826 g (∼81%). The ESI-MS spectrum of the product is shown in Figure 1.

### Synthesis of (Bu_4_N)_2_[SiF_6_⊂{Cu(OH)(pz)}*_n_*] (Cu*_n_*SiF_6_; *n* = 28, 30–32, 34) Using CuF_2_

CuF_2_·2H_2_O (0.534 g, 3.88 mmol), pyrazole (0.264 g, 3.88 mmol), NaOH (0.335 g, 8.38 mmol) and ^*n*^Bu_4_NOH (1 M in H_2_O, 0.290 mg, 0.290 mmol) were stirred together in THF (20 mL) for 4 days. The dark-blue solution was filtered, and the solvent was evaporated. Yield: 0.525 g (∼80%). ESI-MS analysis shows that the product contains **Cu**_**31**_**CO**_**3**_ besides the **Cu***_**n**_***SiF**_**6**_ species (Figure S1).

### Titration of (Bu_4_N)_2_[CO_3_⊂{Cu(OH)(pz)}*_n_*] (Cu*_n_*CO_3_; *n* = 27, 29–31) with Hexafluorosilicic Acid (H_2_SiF_6_)

A 1.0 × 10^–4^ M solution of carbonate nanojars was prepared by dissolving **Cu***_**n**_***CO**_**3**_ (12.1 mg, 2.5 × 10^–6^ mol, based on an average *n* = 29) in CH_3_CN and diluting to volume in a 25 mL volumetric flask. A 5.0 × 10^–2^ M H_2_SiF_6_ solution was prepared by diluting a H_2_SiF_6_ solution (34.2% in H_2_O, 402 μL 1.25 × 10^–3^ mol) with CH_3_CN to volume in a 25 mL volumetric flask. A 5.0 × 10^–3^ M H_2_SiF_6_ solution was prepared by diluting 2.5 mL of the 5.0 × 10^–2^ M H_2_SiF_6_ solution with CH_3_CN to volume in a 25 mL volumetric flask. 1.0 mL of the **Cu***_**n**_***CO**_**3**_ solution was transferred to a dram vial using a 1000 μL micropipette. Twenty μL of the 5.0 × 10^–3^ M H_2_SiF_6_ solution are required per 1.0 mL of the 1.0 × 10^–4^ M **Cu***_**n**_***CO**_**3**_ solution for each molar equivalent. A series of solutions containing 0, 1, 2, 3, 4, 5, 6, 7, 8, 9, 10, 15, 20, 25, 30, 60, and 100 mol equiv of H_2_SiF_6_ was prepared. After the addition of the H_2_SiF_6_, the vial was capped, swirled, and allowed to stand overnight (∼14 h) at ambient conditions. The following day, the solutions were filtered before analysis by ESI-MS (no precipitate formation was observed in any of the solutions).

### Titration of (Bu_4_N)_2_[CO_3_⊂{Cu(OH)(pz)}*_n_*] (Cu*_n_*CO_3_; *n* = 27, 29–31) with Hydrofluoric Acid (HF)

A 1.0 × 10^–4^ M solution of nanojars was prepared by dissolving **Cu***_**n**_***CO**_**3**_ (12.1 mg, 2.5 × 10^–6^ mol, based on an average *n* = 29) in CH_3_CN and diluting to volume in a 25 mL volumetric flask. A 1.0 × 10^–1^ M HF solution was prepared by diluting hydrofluoric acid (40% in H_2_O, 217.5 μL, 5.0 × 10^–3^ mol) with CH_3_CN to volume in a 50 mL volumetric flask. A 5.0 × 10^–3^ M HF solution was prepared by taking 2.5 mL from the 1.0 × 10^–1^ M HF solution and diluting with CH_3_CN to volume in a 50 mL volumetric flask. 0.50 mL of the **Cu***_**n**_***CO**_**3**_ solution was transferred into an Eppendorf vial using a 1000 μL micropipette. Ten μL of the 5.0 × 10^–3^ M HF solution are required per 0.50 mL of the 1.0 × 10^–4^ M **Cu***_**n**_***CO**_**3**_ solution for each molar equivalent. A series of solutions containing 0, 1, 2, 3, 4, 5, 6, 7, 8, 9, 10, 15, 20, 25, 30, 60, and 100 mol equiv of HF was prepared. After the addition of the HF, the vial was capped, swirled, and allowed to stand overnight (∼14 h) at ambient conditions. The following day, the solutions were filtered before analysis by ESI-MS (precipitate formation was observed for samples containing 30, 60, and 100 mol equiv of HF).

### Titration of (Bu_4_N)_2_[CO_3_⊂{Cu(OH)(pz)}*_n_*] (Cu*_n_*CO_3_; *n* = 27, 29–31) with Tetrafluoroboric Acid (HBF_4_)

A 1.0 × 10^–4^ M solution of carbonate nanojars was prepared by dissolving **Cu***_**n**_***CO**_**3**_ (12.1 mg, 2.5 × 10^–6^ mol, based on an average *n* = 29) in CH_3_CN and diluting to volume in a 25 mL volumetric flask. A 5.0 × 10^–2^ M HBF_4_ solution was prepared by diluting tetrafluoroboric acid (48% in H_2_O, 163.3 μL, 1.25 × 10^–3^ mol) with CH_3_CN to volume in a 25 mL volumetric flask. A 5.0 × 10^–3^ M HBF_4_ solution is prepared by taking 2.5 mL from the 5.0 × 10^–2^ M HBF_4_ solution and diluting with CH_3_CN to volume in a 25 mL volumetric flask. 1.0 mL of the **Cu***_**n**_***CO**_**3**_ solution is transferred into a dram vial using a 1000 μL micropipette. Twenty μL of the 5.0 × 10^–3^ M HBF_4_ solution are required per 1.0 mL of the 1.0 × 10^–4^ M **Cu***_**n**_***CO**_**3**_ solution for each molar equivalent. A series of solutions containing 0, 0.5, 1, 1.5, 2, 2.5, 3, 3.5, 4, 4.5, 5, 6, 7, 8, 9, 10, 15, 20, 25, 30, 60, and 100 mol equiv of HBF_4_ was prepared. After the addition of the HBF_4_, the vial was capped, swirled, and allowed to stand overnight (∼14 h) at ambient conditions. The following day, the solutions were filtered before analysis by ESI-MS (no precipitate formation was observed in any of the solutions).

### Mass Spectrometry

Mass spectrometric analysis of the nanojars was performed with a Waters Synapt G1 HDMS instrument, using electrospray ionization (ESI). 10^–4^–10^–5^ M solutions were prepared in CH_3_CN using either solids or aliquots taken from solutions. Samples were infused by a syringe pump at 5 μL/min and nitrogen was supplied as the nebulizing gas at 500 L/h. The electrospray capillary voltage was set to −2.5 or +2.5 kV, respectively, with a desolvation temperature of 110 °C. The sampling and extraction cones were maintained at 40 and 4.0 V, respectively, at 80 °C. Reported *m*/*z* values represent averages of the observed isotopic distributions.

### X-ray Crystallography

Single-crystals of **1** were grown at room temperature by *n*-pentane vapor diffusion into a chlorobenzene solution. Once removed from the mother liquor, the crystals are extremely sensitive to solvent loss at ambient conditions and were quickly mounted under a cryostream (150 K) to prevent decomposition. X-ray diffraction data were collected from a single-crystal mounted atop a MiTeGen micromesh mount under Fomblin oil with a Bruker AXS D8 Quest diffractometer with Photon III C14 charge-integrating and photon counting pixel array detector (CPAD) using a microfocus X-ray tube with multilayer optics for monochromatization with Cu-*K*_α_ (λ = 1.54178 Å) radiation. The data were collected using APEX4,^80^ integrated using SAINT^81^ and scaled and corrected for absorption and other effects using SADABS.^82^ The structure was solved by employing direct methods using ShelXS^83^ or ShelXT^84^ and refined by full-matrix least-squares on *F*^2^ using ShelXL.^85^ Refinement details and a thermal ellipsoid plot (Figure S6) are provided in the Supporting Information. Crystallographic figures were generated using CrystalMaker,^86^ and supramolecular features (angles and distances) were measured using OLEX2.^87^

## Supporting Information Available

Additional data for mass spectrometry, X-ray crystallography and ^1^H NMR spectroscopy (PDF)
Crystallographic data (CIF)

## Supplementary Material

ao4c08535_si_001.pdf

ao4c08535_si_002.cif
