# Supplementary material for: Where Fluoride Is Present, Hexafluorosilicate Might Be Encountered: Supramolecular Binding of the SiF62– Anion by Nanojars
Source: ACS Omega. 2024 Oct 18;9(43):43986–97. doi: 10.1021/acsomega.4c08535 (PMC11525531; doi:10.1021/acsomega.4c08535)
Supplement: Supplementary file 1 — ao4c08535_si_001.pdf [file ao4c08535_si_001.pdf]

Supporting Information for

Where Fluoride Is Present, Hexafluorosilicate Might Be Encountered: Supramolecular Binding of the SiF_6^{2-} Anion by Nanojars

Wisam A. Al Isawi,[a] Matthias Zeller[b] and Gellert Mezei[a*]

[a] *Department of Chemistry, Western Michigan University, Kalamazoo, Michigan 49008, USA*

[b] *Department of Chemistry, Purdue University, West Lafayette, Indiana 47907, USA*

[*] Corresponding author. Email: gellert.mezei@wmich.edu

CONTENTS **PAGE**

1. Mass spectrometric data (Figures S1−S5) S2−S6
2. X-ray crystallographic data and refinement details (Figure S6 and Tables S1−S8) S7−S14
3. NMR spectroscopic data (Tables S9 and S10, Figures S7−S9) S15−S18

1. MASS SPECTROMETRIC DATA

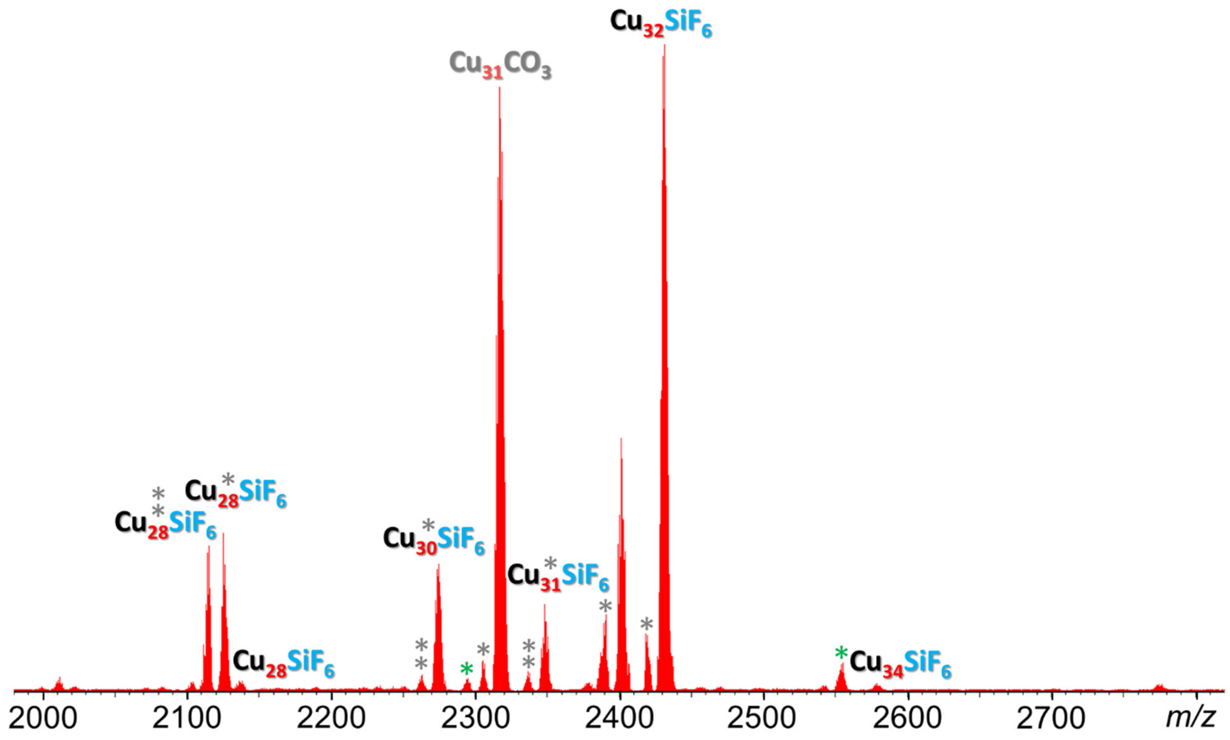

Figure S1. ESI-MS(−) spectrum (in CH_3CN) of the nanojar mixture obtained from CuF_2, pyrazole, NaOH and Bu_4NOH in THF. Grey asterisks denote singly or doubly formate-substituted species, wherein one or two pz^- moieties are exchanged by $HCOO^-$. Nanojars in general are extremely sensitive to even traces of formic acid in the mass spectrometer, which is commonly used as an additive to improve peak shapes and to promote ionization. Therefore, substituted species such as $[SiF_6^{2-}\subset\{Cu_{28}(OH)_{28}(pz)_{28-y}(HCOO)_y\}]$ (y = 1, *m/z* 2127; y = 2, *m/z* 2116) are sometimes observed in the mass spectra of nanojars, at 11 *m/z* units less than the parent species. If larger amounts of formic acid are present, only formate-substituted species are observed in the case of more vulnerable species, such as **$Cu_{28}SiF_6$**, **$Cu_{30}SiF_6$** and **$Cu_{31}SiF_6$**. Green asterisks denote similar species where the pz^- moiety is replaced by F^-, observed at 24 *m/z* units less than the parent species.

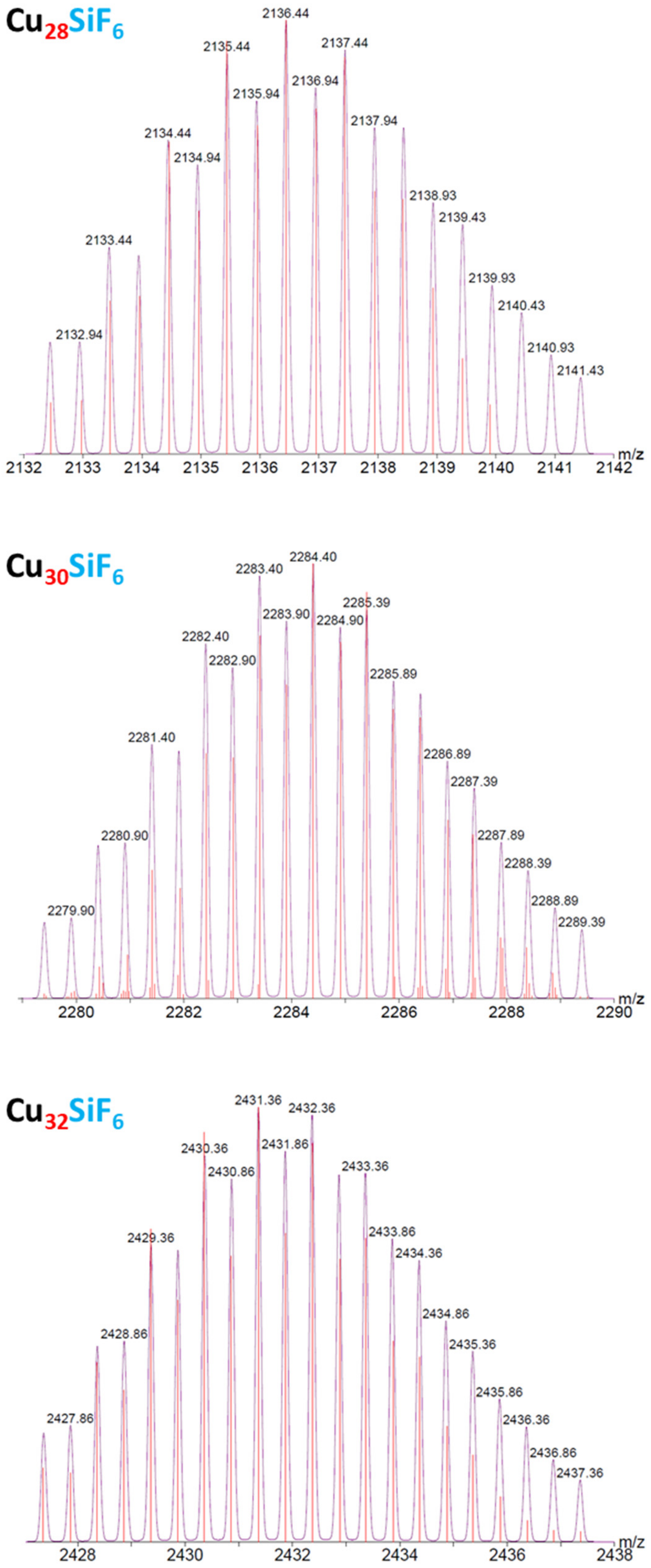

Figure S2. Isotopic distributions observed (centroid) and predicted (continuum) for the major SiF_6-nanojars.

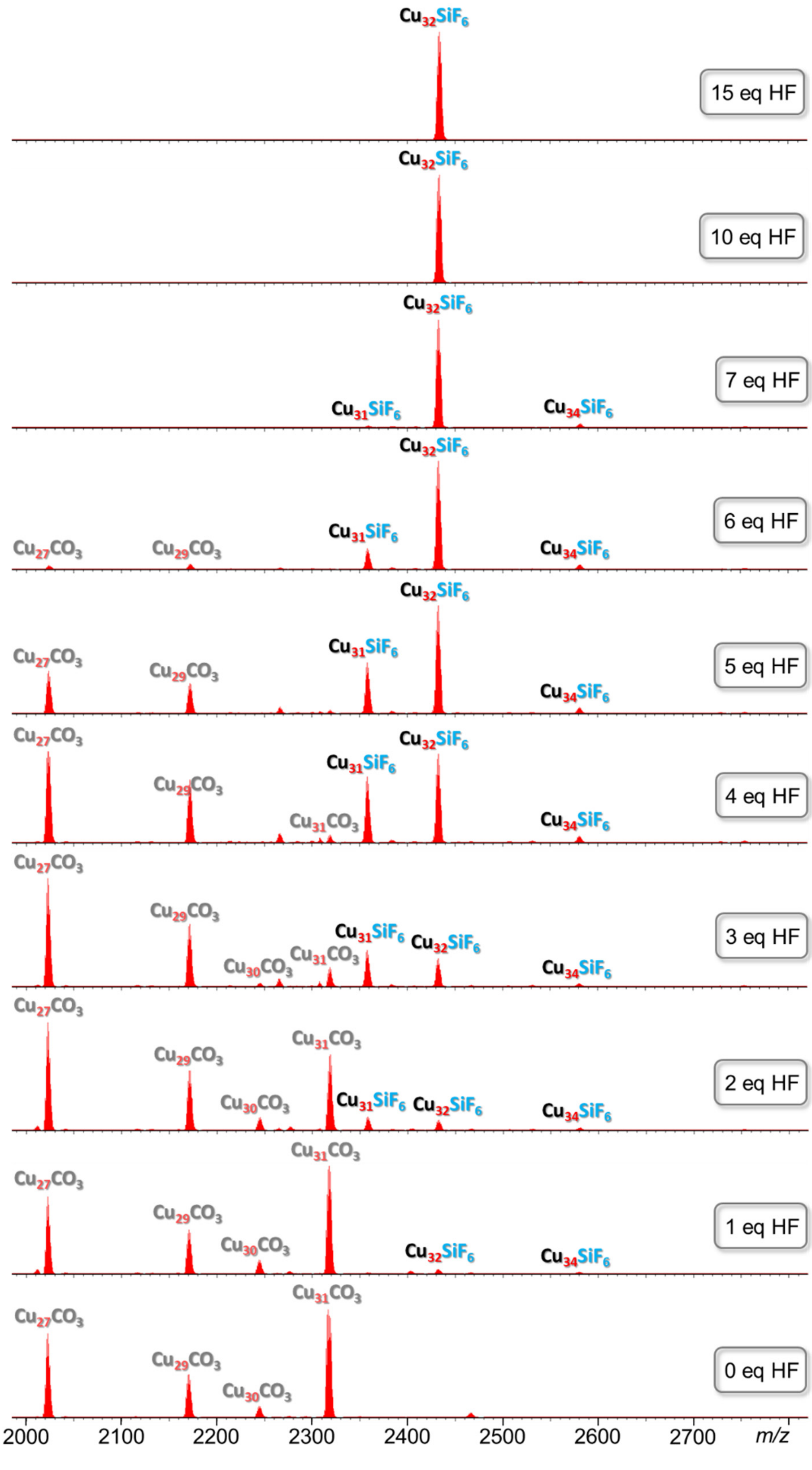

Figure S3. ESI-MS(−) spectra in CH_3CN of $\mathbf{Cu_nCO_3}$ (n = 27, 29−31) nanojars with varying amounts of added HF.

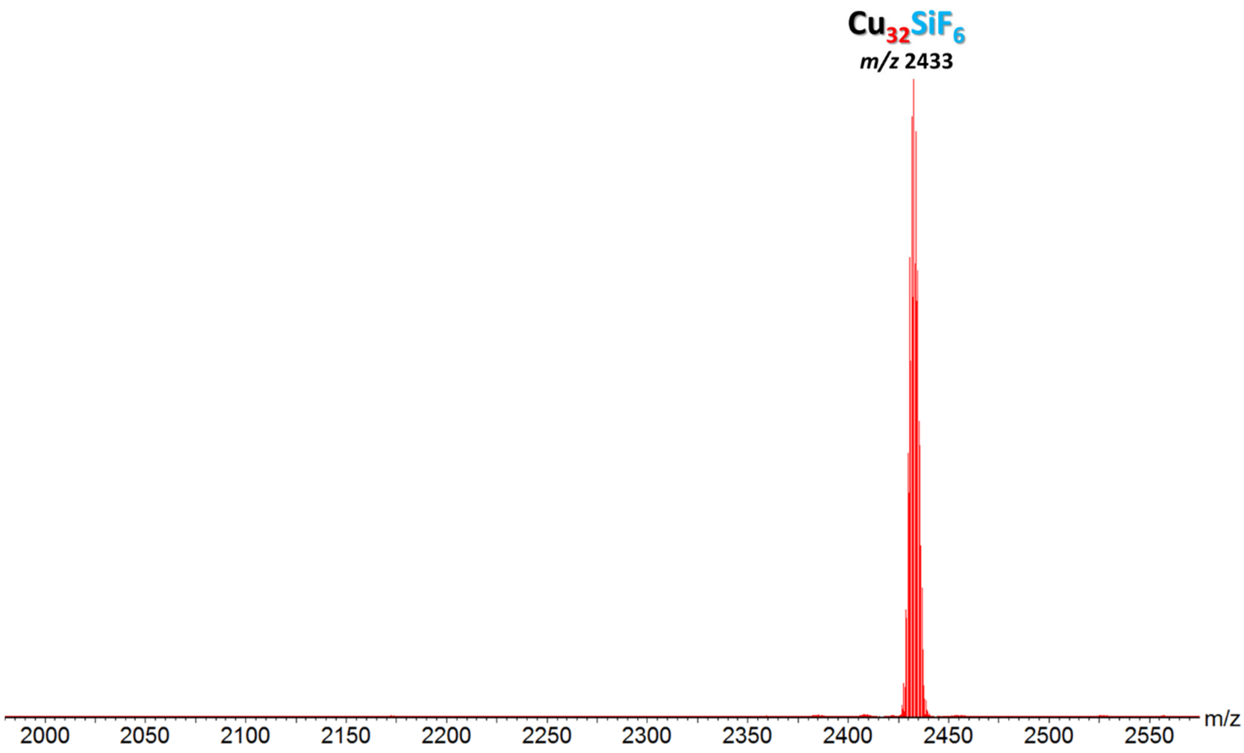

Figure S4. ESI-MS(−) spectrum of $[SiF_6\subset\{Cu(OH)(pz)\}_{32}]^{2-}$ synthesized by combining $[CO_3\subset\{Cu(OH)(pz)\}_n]^{2-}$ (n = 27, 29−31) with 8 equivalents of HF in CH_3CN.

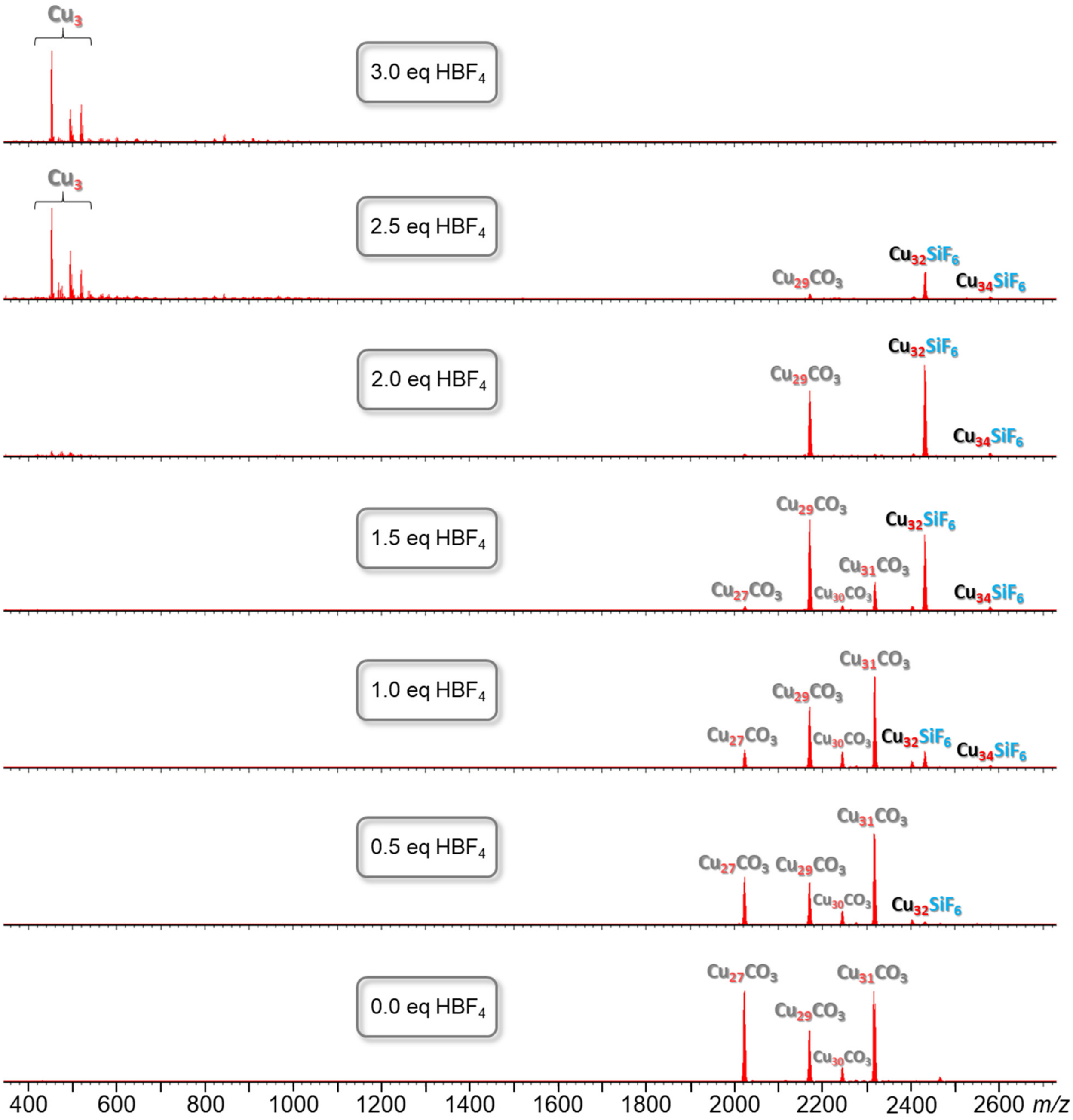

Figure S5. ESI-MS(−) spectra in CH_3CN of **Cu_nCO_3** (n = 27, 29−31) nanojars with varying amounts of added HBF_4.

2. X-RAY CRYSTALLOGRAPHIC DATA

1: C–H hydrogen atoms were placed in idealized positions and refined using the riding model. Hydroxyl H atom positions were refined and O–H distances were restrained to 0.84(2) Å. The SiF_6^{2-} anion was refined as disordered by a 45° rotation. The Si and F atoms along the rotation axis were constrained to have pairwise identical ADPs. The two disordered moieties were restrained to have similar geometries. U_{ij} components of ADPs for disordered atoms closer to each other than 2.0 Å were restrained to be similar. Subject to these conditions the occupancy ratio refined to 0.860(5)/0.140(5).

Bu_4N^+ cations are disordered. One cation is located in a general position and whole cation disorder was refined. Two other cations are located on two-fold axes. One was refined as disordered around the two-fold axis with two half-occupied symmetry-equivalent moieties. The other was refined as two-fold symmetric but disordered over two moieties slightly shifted along the axis. The N–C bond lengths of all cations were restrained to be similar in length. Butyl groups of major moieties were restrained to have similar geometries. Minor moieties were restrained to have similar geometries as major moieties. U_{ij} components of ADPs for disordered atoms closer to each other than 2.0 Å were restrained to be similar. Subject to these conditions the occupancy ratio refined to 0.436(5)/0.564(5) for the cation in the general position, and to 0.652(7)/0.348(7) for the cation shifted along the two-fold axis.

Heavily disordered chlorobenzene molecules are present, which are disordered with the cations and among themselves, and with a single partially-occupied *n*-pentane molecule. Disorder is general as well as symmetry imposed by two-fold axes. Disordered chlorobenzene moieties were restrained to have similar geometries and were restrained to be close to planar. For several minor moieties, the benzene rings were constrained to resemble ideal hexagons with C–C bond lengths of 1.39 Å. The bond distances of the *n*-pentane molecule were restrained to expected target values. U_{ij} components of ADPs for disordered atoms closer to each other than 2.0 Å were restrained to be similar. Subject to these conditions the occupancy rates refined to the values given in the CIF tables.

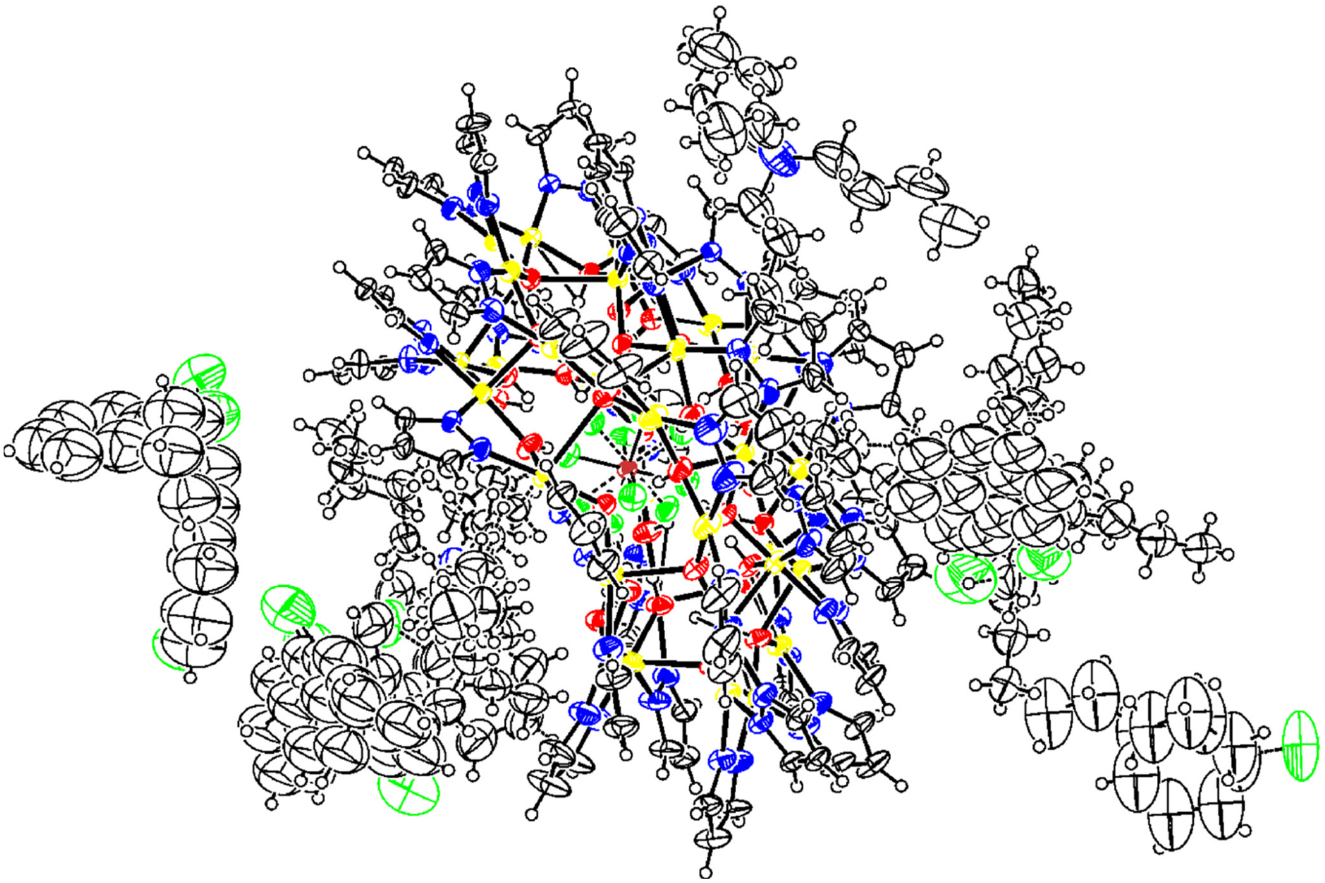

Figure S6. Thermal ellipsoid plot of the crystal structure of **1**.

Table S1. Crystallographic data for **1**.

	**1**
Formula (sum)	$C_{152.396}H_{222.149}Cl_{3.719}Cu_{32}F_6N_{66}O_{32}Si$
Formula (moiety)	$C_{96}H_{128}Cu_{32}N_{64}O_{32}$, F_6Si, 2($C_{16}H_{36}N$), 3.719(C_6H_5Cl), 0.416(C_5H_{12})
FW (g·mol^{-1})	5798.34
Crystal system	Tetragonal
Space group	$I\bar{4}2d$ (No. 122)
a (Å)	41.6926(8)
b (Å)	41.6926(8)
c (Å)	49.6073(14)
α (deg)	90.000
β (deg)	90.000
γ (deg)	90.000
V (Å^3)	86231(4)
Z	16
D_{calc} (g·cm^{-3})	1.786
μ (mm^{-1})	4.438
θ range (deg)	2.119–79.412
Reflns collected	195411
R_{int}	0.0418
Obsd reflns [$I > 2\sigma(I)$]	38235
Data/restraints/parameters	41352/3985/3532
GOF (on F^2)	1.094
R factors [$I > 2\sigma(I)$]	R_1 = 0.0338<br>wR_2 = 0.0824
R factors (all data)	R_1 = 0.0388<br>wR_2 = 0.0862
Maximum peak/hole (e·Å^{-3})	0.753/−0.486
CCDC number	2359778

Table S2. Bond lengths (Å) and angles (°), Cu···O distances shorter than the sum of the van der Waals radii of Cu and O (2.92 Å) and H-bonding (with O···O distances shorter than 3.20 Å and O···F distances shorter than 3.35 Å, based on the major SiF_6^{2-} component) in **1**.

	**$Cu_{32}SiF_6$ (1)**
Cu–O within Cu_n rings	1.891(5)–1.959(4)<br>avg: 1.929(5)
Cu–N within Cu_n rings	1.946(6)–2.011(5)<br>avg: 1.972(6)
N–Cu–O (*trans*) within Cu_{14} ring	165.0(2)–175.5(2)<br>avg: 170.1(2)
N–Cu–O (*trans*) within Cu_{10} ring	167.0(2)–179.1(3)<br>avg: 174.0(2)
N–Cu–O (*trans*) within Cu_8 ring	166.1(3)–177.4(2)<br>avg: 171.9(2)
Average of all N–Cu–O (trans) angles	*172.0(2)*
N–Cu–O (*cis*) within Cu_{14} ring	85.1(2)–88.1(2)<br>avg: 87.0(2)
N–Cu–O (*cis*) within Cu_{10} ring	84.0(2)–87.6(2)<br>avg: 85.3(2)
N–Cu–O (*cis*) within Cu_8 ring	83.5(2)–87.5(2)<br>avg: 84.9(2)
Average of all N–Cu–O (cis) angles	*85.7(2)*
Cu···Cu distances in Cu_{14} ring	3.1660(14)–3.3852(12)<br>avg: 3.287(2)
Cu···Cu distances in Cu_{10} ring	3.1257(14)–3.3575(14)<br>avg: 3.277(2)
Cu···Cu distances in Cu_8 ring	3.2630(13)–3.3883(12)<br>avg: 3.337(2)
Average of all Cu···Cu distances in Cu_n rings	*3.300(2)*
Cu···O between Cu_{10} and Cu_{14} rings	2.358(4)–2.833(5)<br>(6 interactions)<br>avg: 2.548(5)
Cu···O between Cu_8 and Cu_{14} rings	2.342(4)–2.900(5)<br>(8 interactions)<br>avg: 2.538(5)
Average of all Cu···O interactions between Cu_n rings	*2.543(5)*<br>*(14 interactions)*
H-bonded O···O distances between Cu_{10} and Cu_{14} rings	2.746(6)–2.822(6)<br>(6 interactions)<br>avg: 2.789(6)
H-bonded O···O distances between Cu_8 and Cu_{14} rings	2.756(6)–2.887(6)<br>(6 interactions)<br>avg: 2.822(6)
Average of all H-bonded O···O distances between Cu_n rings	*2.805(6)*<br>*(12 interactions)*
H-bonded O···F distances between Cu_n rings and SiF_6^{2-}	2.740(6)–3.228(12)<br>(18 interactions)<br>avg: 2.933(6)

Table S3. Comparison of the dihedral, twist and fold angles (°) between adjacent pyrazolate moieties in **1**.

	DIHEDRAL ANGLE	TWIST ANGLE	FOLD ANGLE	CENTROID-CENTROID DISTANCE	
				D. A. > 35°	D. A. < 35°
**$Cu_{32}SiF_6$ (1)** Cu_{14}-ring	42.8(4)–64.0(3) avg: 53.3(4)	41.0(4)–63.1(3) avg: 53.3(4)	3.5(4)–52.4(10) avg: 19.9(4)	4.683(5)–4.936(7) avg. of 14: 4.796(5)	–
**$Cu_{32}SiF_6$ (1)** Cu_{10}-ring	21.6(3)–57.0(3) avg: 41.6(3)	12.0(4)–54.5(4) avg: 40.0(4)	0.9(3)–23.9(4) avg: 15.9(4)	4.825(4)–4.889(5) avg of 7: 4.851(5)	4.909(4)–5.147(5) avg of 3: 5.050(5)
**$Cu_{32}SiF_6$ (1)** Cu_8-ring	23.0(3)–63.1(3) avg: 40.1(3)	0.5(3)–60.9(4) avg: 30.3(4)	0.1(5)–49.0(6) avg: 26.8(5)	4.832(4)–4.889(5) avg of 4: 4.854(5)	5.018(5)–5.101(4) avg of 4: 5.056(5)
Avg. **$Cu_{32}SiF_6$ (1)**	*45.0(4)*	*41.2(4)*	*20.9(4)*	*4.870(4)*	*5.053(5)*

Table S4. Comparison of the dihedral, twist and fold angles (°) between pyrazolate moieties and adjacent Cu–O–Cu units in **1**.

	DIHEDRAL ANGLE	TWIST ANGLE	FOLD ANGLE
**$Cu_{32}SiF_6$ (1)** Cu_{14}-ring	7.3(5)–53.8(3) avg: 40.4(3)	1.5(3)–12.7(2) avg: 6.1(3)	4.8(4)–58.0(7) avg: 40.4(4)
**$Cu_{32}SiF_6$ (1)** Cu_{10}-ring	24.9(4)–67.1(4) avg: 49.1(4)	0.6(4)–8.2(2) avg: 3.2(4)	24.6(4)–67.0(4) avg: 49.6(4)
**$Cu_{32}SiF_6$ (1)** Cu_8-ring	24.7(4)–65.7(3) avg: 47.6(4)	0.8(3)–9.3(4) avg: 3.5(4)	11(2)–65.7(3) avg: 45.3(3)
Avg. **$Cu_{32}SiF_6$ (1)**	*45.7(4)*	*4.3(4)*	*45.1(4)*

Table S5. Comparison of the deviations (Å) of Cu atoms in different Cu_x rings from the Cu_x mean-planes in **1**.

	**Deviation from Cu_x mean-plane**
**$Cu_{32}SiF_6$ (1)** Cu_{14}-ring	0.010–1.543 avg: 0.887
**$Cu_{32}SiF_6$ (1)** Cu_{10}-ring	0.048–0.940 avg: 0.644
**$Cu_{32}SiF_6$ (1)** Cu_8-ring	0.331–0.592 avg: 0.460
Avg. **$Cu_{32}SiF_6$ (1)**	*0.664*

Table S6. Copper coordination geometry indexes, $\tau_4 = (360 - \beta - \alpha)/141$ and $\tau_5 = (\beta - \alpha)/60$ in different Cu_x rings in **1** (where β and α are the two largest angles in the four- or five-coordinate species. Average values are shown in green for the individual Cu_x rings and in orange for the whole nanojar.

	β	α	τ_4	τ_5
**Cu1**	177.3	166.1	0.12	0.19
**Cu2**	176.5	168.8	0.10	0.13
**Cu3**	174.0	170.6	0.11	0.06
**Cu4**	172.84	168.0	0.14	0.08
**Cu5**	176.16	166.7	0.12	0.16
**Cu6**	177.4	169.4	0.09	0.13
**Cu7**	175.5	170.4	0.10	0.08
**Cu8**	174.5	166.6	0.13	0.13
**Cu_8-ring**			**0.11**	**0.12**
**Cu9**	171.4	165.0	0.17	0.11
**Cu10**	175.5	174.5	0.07	0.02
**Cu11**	171.1	170.0	0.13	0.02
**Cu12**	168.2	166.1	0.18	0.03
**Cu13**	169.0	168.0	0.16	0.02
**Cu14**	173.6	170.6	0.11	0.05
**Cu15**	172.9	168.6	0.13	0.07
**Cu16**	172.3	165.0	0.16	0.12
**Cu17**	174.2	173.9	0.08	0.00
**Cu18**	171.0	169.7	0.14	0.02
**Cu19**	169.4	166.5	0.17	0.05
**Cu20**	168.8	166.7	0.17	0.04
**Cu21**	172.5	170.5	0.12	0.03
**Cu22**	170.3	167.9	0.15	0.04
**Cu_{14}-ring**			**0.14**	**0.04**
**Cu23**	179.1	176.5	0.03	0.04
**Cu24**	173.1	169.7	0.12	0.06
**Cu25**	176.1	175.5	0.06	0.01
**Cu26**	171.4	169.7	0.13	0.03
**Cu27**	175.7	173.3	0.08	0.04
**Cu28**	178.4	178.0	0.03	0.01
**Cu29**	171.7	170.8	0.12	0.01
**Cu30**	175.2	173.0	0.08	0.04
**Cu31**	174.8	167.0	0.13	0.13
**Cu32**	176.8	174.1	0.06	0.05
**Cu_{10}-ring**			**0.09**	**0.04**
**Cu_{32}**			**0.11**	**0.07**

Table S7. Selected bond lengths (Å) for **1** (Cu1–Cu8: Cu_8-ring; Cu9–Cu22: Cu_{14}-ring; Cu23–Cu32: Cu_{10}-ring; F1–F6, F1b–F6b: SiF_6^{2-} anion disordered over two positions in 86/14 ratio).

Si1–F1 1.680(6)	Cu6–N10 1.986(5)	Cu15–N28 1.974(6)	Cu24–N47 2.000(6)
Si1–F2 1.707(6)	Cu7–O6 1.909(4)	Cu16–O16 1.930(4)	Cu24–O10 2.372(5)
Si1–F3 1.686(6)	Cu7–O7 1.926(4)	Cu16–O15 1.941(4)	Cu25–O25 1.897(5)
Si1–F4 1.694(5)	Cu7–N13 1.972(5)	Cu16–N30 1.950(5)	Cu25–O24 1.925(4)
Si1–F5 1.659(5)	Cu7–N12 1.977(6)	Cu16–N31 1.971(5)	Cu25–N49 1.959(5)
Si1–F6 1.664(6)	Cu8–O7 1.949(4)	Cu17–O17 1.922(4)	Cu25–N48 1.973(6)
Si1b–F1b 1.68(2)	Cu8–O8 1.959(4)	Cu17–O16 1.939(4)	Cu26–O25 1.932(4)
Si1b–F2b 1.70(2)	Cu8–N15 1.982(6)	Cu17–N32 1.950(5)	Cu26–O26 1.942(4)
Si1b–F3b 1.74(2)	Cu8–N14 2.011(5)	Cu17–N33 1.974(5)	Cu26–N50 1.975(5)
Si1b–F4b 1.69(2)	Cu8–O21 2.369(5)	Cu18–O18 1.920(4)	Cu26–N51 2.005(5)
Si1b–F5b 1.70(2)	Cu9–O22 1.931(5)	Cu18–O17 1.925(4)	Cu26–O13 2.425(4)
Si1b–F6b 1.67(2)	Cu9–O9 1.934(5)	Cu18–N34 1.962(6)	Cu27–O27 1.915(4)
Cu1–O8 1.938(5)	Cu9–N44 1.960(6)	Cu18–N35 1.962(6)	Cu27–O26 1.918(4)
Cu1–O1 1.943(4)	Cu9–N17 1.962(6)	Cu19–O18 1.912(4)	Cu27–N52 1.952(5)
Cu1–N16 1.987(5)	Cu10–O10 1.925(5)	Cu19–O19 1.924(5)	Cu27–N53 1.962(5)
Cu1–N1 1.999(6)	Cu10–O9 1.937(5)	Cu19–N37 1.959(6)	Cu28–O27 1.914(4)
Cu2–O2 1.925(5)	Cu10–N19 1.965(6)	Cu19–N36 1.971(6)	Cu28–O28 1.917(4)
Cu2–O1 1.937(5)	Cu10–N18 1.969(6)	Cu20–O20 1.905(4)	Cu28–N55 1.948(5)
Cu2–N3 1.981(6)	Cu11–O10 1.928(5)	Cu20–O19 1.922(4)	Cu28–N54 1.964(5)
Cu2–N2 1.996(6)	Cu11–O11 1.931(5)	Cu20–N39 1.963(5)	Cu29–O28 1.943(4)
Cu3–O2 1.896(5)	Cu11–N20 1.965(7)	Cu20–N38 1.965(6)	Cu29–O29 1.958(4)
Cu3–O3 1.926(4)	Cu11–N21 1.972(7)	Cu21–O20 1.928(4)	Cu29–N57 1.999(6)
Cu3–N4 1.955(6)	Cu12–O11 1.904(5)	Cu21–O21 1.932(4)	Cu29–N56 2.005(5)
Cu3–N5 1.959(5)	Cu12–O12 1.929(5)	Cu21–N41 1.951(6)	Cu29–O17 2.358(4)
Cu4–O4 1.950(4)	Cu12–N23 1.965(7)	Cu21–N40 1.971(5)	Cu30–O30 1.891(5)
Cu4–O3 1.957(4)	Cu12–N22 1.985(7)	Cu22–O21 1.925(4)	Cu30–O29 1.917(4)
Cu4–N7 1.981(5)	Cu13–O13 1.911(4)	Cu22–O22 1.942(4)	Cu30–N59 1.946(6)
Cu4–N6 2.009(5)	Cu13–O12 1.935(5)	Cu22–N43 1.958(6)	Cu30–N58 1.953(6)
Cu4–O14 2.342(4)	Cu13–N24 1.962(6)	Cu22–N42 1.959(6)	Cu31–O30 1.927(5)
Cu5–O4 1.940(4)	Cu13–N25 1.974(6)	Cu23–O23 1.908(5)	Cu31–O31 1.942(5)
Cu5–O5 1.947(4)	Cu14–O13 1.931(4)	Cu23–O32 1.909(5)	Cu31–N60 1.979(6)
Cu5–N8 1.992(5)	Cu14–O14 1.943(4)	Cu23–N64 1.952(7)	Cu31–N61 1.989(6)
Cu5–N9 2.004(5)	Cu14–N27 1.956(5)	Cu23–N45 1.953(7)	Cu32–O32 1.923(5)
Cu5–O16 2.415(4)	Cu14–N26 1.974(5)	Cu24–O23 1.938(5)	Cu32–O31 1.931(5)
Cu6–O6 1.910(4)	Cu15–O15 1.931(4)	Cu24–O24 1.953(4)	Cu32–N62 1.961(6)
Cu6–O5 1.945(4)	Cu15–O14 1.938(4)	Cu24–N46 1.985(6)	Cu32–N63 1.974(7)
Cu6–N11 1.980(5)	Cu15–N29 1.954(6)		

Table S8. Hydrogen bonding data for **1** (O1– O8: Cu_8-ring; O9–O22: Cu_{14}-ring; O23–O32: Cu_{10}-ring; F1–F6, F1b–F6b: SiF_6^{2-} anion disordered over two positions in 86/14 ratio).

D*—H···*A	***D*—H (Å)**	**H···*A* (Å)**	***D*···*A* (Å)**	***D*—H···*A* (°)**
O1—H1O···F2	0.84(3)	2.18(4)	2.998(10)	165(8)
O1—H1O···F2b	0.84(3)	2.12(6)	2.95(5)	169(8)
O2—H2O···F2	0.81(3)	2.55(6)	3.228(12)	141(9)
O2—H2O···F3	0.81(3)	2.17(5)	2.908(7)	151(9)
O2—H2O···F2b	0.81(3)	2.41(8)	3.10(5)	144(9)
O2—H2O···F3b	0.81(3)	2.30(5)	3.05(3)	154(9)
O3—H3O···F3	0.82(3)	1.95(4)	2.752(6)	164(8)
O3—H3O···F6b	0.82(3)	2.21(5)	2.98(2)	155(7)
O4—H4O···F3	0.84(3)	2.15(4)	2.971(6)	164(8)
O4—H4O···F6b	0.84(3)	2.51(7)	3.14(3)	133(7)
O5—H5O···F4	0.82(3)	2.28(3)	3.083(10)	166(8)
O6—H6O···F1	0.82(3)	2.18(5)	2.920(6)	149(8)
O6—H6O···F3b	0.82(3)	2.20(5)	2.99(3)	162(8)
O7—H7O···F1	0.81(3)	2.01(5)	2.740(6)	151(8)
O7—H7O···F1b	0.81(3)	2.00(4)	2.81(2)	174(8)
O8—H8O···F1	0.83(3)	2.18(4)	2.987(6)	163(8)
O8—H8O···F1b	0.83(3)	2.36(6)	3.08(3)	146(8)
O9—H9O···O23	0.84(3)	1.99(3)	2.822(7)	173(9)
O10—H10O···O1	0.84(3)	1.97(3)	2.801(7)	173(9)
O11—H11O···O24	0.82(3)	1.97(3)	2.785(6)	174(9)
O12—H12O···O2	0.83(3)	2.58(5)	3.340(7)	155(9)
O13—H13O···O3	0.83(3)	1.94(3)	2.771(6)	175(8)
O14—H14O···O26	0.84(3)	1.94(4)	2.747(6)	163(8)
O15—H15O···O4	0.84(3)	2.09(4)	2.887(6)	159(7)
O16—H16O···O28	0.84(3)	1.97(3)	2.791(6)	166(8)
O17—H17O···O5	0.83(3)	2.11(5)	2.858(6)	151(7)
O17—H17O···N9	0.83(3)	2.59(6)	3.253(6)	138(7)
O17—H17O···N10	0.83(3)	2.59(5)	3.283(7)	142(7)
O18—H18O···O29	0.83(3)	1.96(3)	2.796(6)	175(8)
O20—H20O···O7	0.83(3)	1.95(4)	2.756(6)	164(8)
O21—H21O···O31	0.81(3)	2.01(4)	2.793(7)	163(9)
O22—H22O···O8	0.83(3)	2.04(3)	2.859(7)	170(9)
O23—H23O···F2	0.82(3)	2.10(4)	2.900(10)	163(9)
O23—H23O···F2b	0.82(3)	2.25(6)	3.04(4)	161(9)
O24—H24O···F2	0.83(3)	2.08(4)	2.875(9)	160(8)
O24—H24O···F2b	0.83(3)	2.15(6)	2.95(4)	162(8)
O25—H25O···F6	0.82(3)	2.35(4)	3.126(6)	159(9)
O25—H25O···F6b	0.82(3)	2.45(5)	3.23(3)	161(9)
O26—H26O···F6	0.80(3)	2.04(3)	2.830(6)	169(8)
O26—H26O···F6b	0.80(3)	1.93(5)	2.68(2)	156(8)
O27—H27O···F6	0.82(3)	2.24(6)	2.912(6)	139(7)
O27—H27O···F6b	0.82(3)	2.49(5)	3.25(3)	154(8)
O28—H28O···F4	0.81(3)	2.13(3)	2.928(9)	167(8)
O29—H29O···F4	0.83(3)	2.09(3)	2.910(8)	169(8)
O30—H30O···F1b	0.81(3)	2.55(8)	3.18(3)	136(9)
O31—H31O···F5	0.80(3)	2.09(4)	2.854(6)	159(9)
O31—H31O···F1b	0.80(3)	2.10(6)	2.84(3)	154(9)
O32—H32O···F5	0.83(3)	2.08(4)	2.873(7)	160(10)
O32—H32O···F5b	0.83(3)	2.61(6)	3.34(3)	147(9)

3. NMR SPECTROSCOPIC DATA

Table S9. Variable-temperature ^{1}H NMR chemical shifts (ppm) in DMSO-d_6 of **Cu_nSiF_6** nanojars. Missing values are due to lack of significant amounts of a particular species in the mixture and/or to inability of unambiguous assignment due to overlap or excessive broadening.

NANOJAR	25 °C	30 °C	40 °C	50 °C	60 °C	70 °C	80 °C	90 °C	100 °C	110 °C	120 °C	130 °C	140 °C	150 °C
**Cu_{28} (6+12+10)**														
Cu_{10} ring, pz-4-*H*	34.91	34.72	34.34	33.98	33.62	33.27	32.94	32.62	32.30	31.98	–	–	–	–
Cu_{10} ring, pz-3,5-H_2	30.75	30.59	30.27	29.96	29.65	29.36	29.07	28.78	~28.5	28.23	–	–	–	–
Cu_6 ring, pz-4-*H*	34.42	34.43	34.41	34.36	34.27	34.17	34.04	33.89	33.72	33.55	–	–	–	–
Cu_6 ring, pz-3,5-H_2	31.14	31.14	31.12	31.07	31.00	30.91	30.79	30.67	30.55	30.43	–	–	–	–
Cu_{12} ring, pz-4-*H(9)*	29.17	29.20	29.24	29.27	29.29	29.30	29.31	29.29	29.27	29.24	–	–	–	–
Cu_{12} ring, pz-4-*H(6)*	27.26	27.28	27.31	27.33	27.33	27.33	27.32	27.30	27.26	27.22	–	–	–	–
Cu_{12} ring, pz-3,5-H_2*(9)*	23.37	23.38	23.39	23.39	23.38	23.37	23.35	23.32	23.29	23.24	–	–	–	–
Cu_{12} ring, pz-3,5-H_2*(6)*	23.26	23.27	23.29	23.30	23.30	23.30	23.29	23.27	23.25	23.21	–	–	–	–
Cu_{12} ring, O*H(6)*	−28.50	−28.47	−28.39	−28.30	−28.21	−28.11	−27.99	−27.88	−27.78	~−27.7	–	–	–	–
Cu_{12} ring, O*H(9)*	−42.29	−42.37	−42.43	−42.47	~−42.5	~−42.4	~−42.2	~−41.9	~−41.7	~−41.5	–	–	–	–
Cu_6 ring, O*H*	− 44.19	−44.21	~−44.2	~−44.2	−44.13	−44.06	−43.99	−43.86	−43.70	~−43.5	–	–	–	–
Cu_{10} ring, O*H*	−45.54	−45.14	~−44.4	−43.66	−41.98	−42.33	−41.74	−41.15	−40.60	−40.07	–	–	–	–
**Cu_{32} (8+14+10)**														
Cu_{10} ring, pz-4-*H*	–	–	–	–	–	31.63	~31.4	~31.3	~31.2	~31.0	~30.9	30.74	30.59	30.45
Cu_{10} ring, pz-3,5-H_2	–	–	–	–	–	27.91	27.78	27.66	27.53	27.39	27.25	27.11	26.97	26.84
Cu_8 ring, pz-4-*H*	–	–	–	–	–	~31.5	~31.4	~31.3	~31.2	~31.0	~30.9	~30.8	30.65	30.52
Cu_8 ring, pz-3,5-H_2	–	–	–	–	–	~28.1	28.04	27.94	27.85	27.75	27.64	27.53	27.42	27.31
Cu_{14} ring, pz-4-*H*	–	–	–	–	–	28.48	28.50	28.53	28.54	28.54	28.53	28.52	28.49	28.46
Cu_{14} ring, pz-3,5-H_2	–	–	–	–	–	22.62	22.63	22.63	22.62	22.61	22.58	22.56	22.52	22.49
Cu_{14} ring, O*H*	–	–	–	–	–	–	–	~−28.7	−28.66	−28.63	−28.62	−28.60	−28.54	−28.47
Cu_8 ring, O*H*	–	–	–	–	–	–	–	~−32.3	−31.99	−31.64	−31.33	−31.03	−30.73	−30.46
Cu_{10} ring, O*H*	–	–	–	–	–	–	–	~−38.0	−37.50	−37.05	−36.78	−36.51	−36.20	−35.88

Table S10. Variable-temperature ^{19}F NMR chemical shifts (ppm) of nanojars **Cu_nSiF_6** (n = 28 and 32) in DMSO-d_6 (referenced to $C_6H_5CF_3$ as internal standard).

NANOJAR	25 °C	30 °C	40 °C	50 °C	60 °C	70 °C	80 °C	90 °C	100 °C	25 °C (after cooling)
**Cu_{28} (6+12+10)**	−26.68	−26.88	−27.31	−27.77	−28.25	−28.73	−29.22	−29.71	−30.19	−26.68
**Cu_{32} (8+14+10)**	−45.03	−44.70	−44.09	−43.57	−43.13	−42.75	−42.43	−42.17	−41.96	−45.03

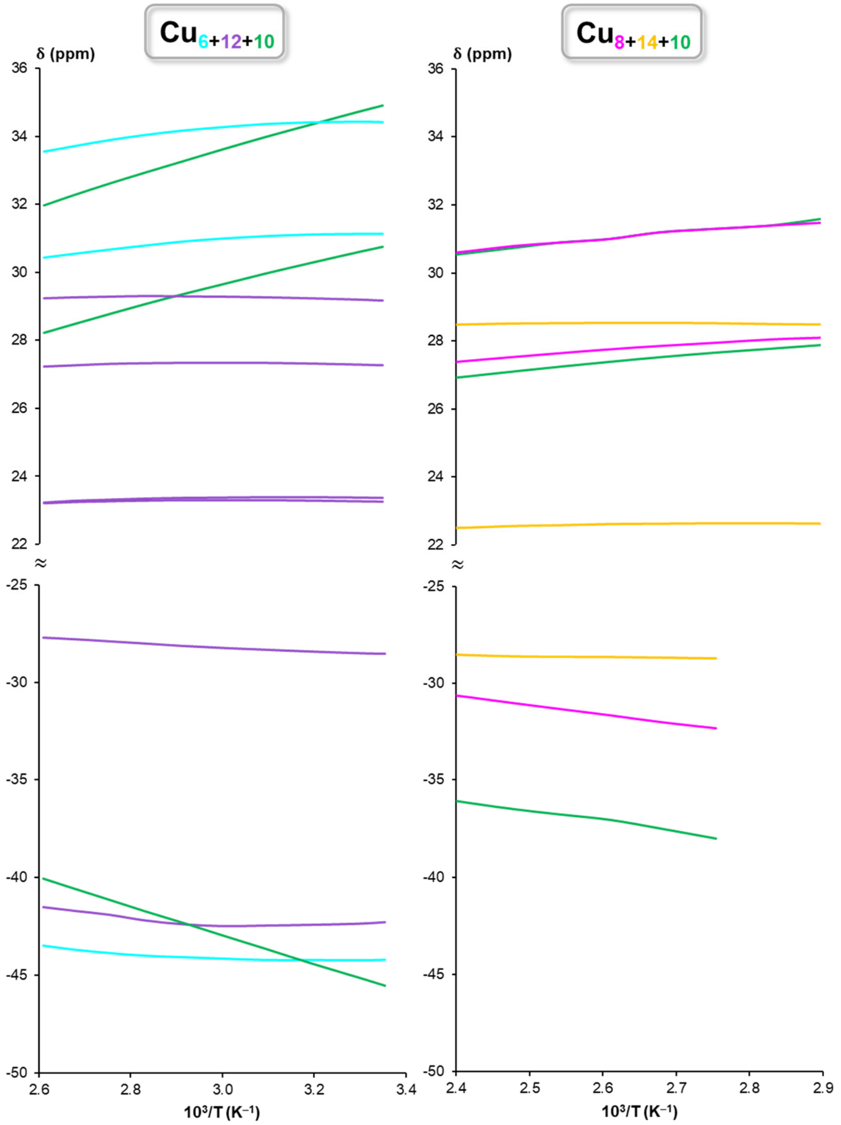

Figure S7. Curie plots for different Cu_x ring protons in the **$Cu_{28}SiF_6$** and **$Cu_{32}SiF_6$** nanojars in DMSO-*d*$_6$, illustrating the influence of the size of the ring on chemical shift, as well as the difference in chemical shift for the same Cu_x-ring in different nanojars.

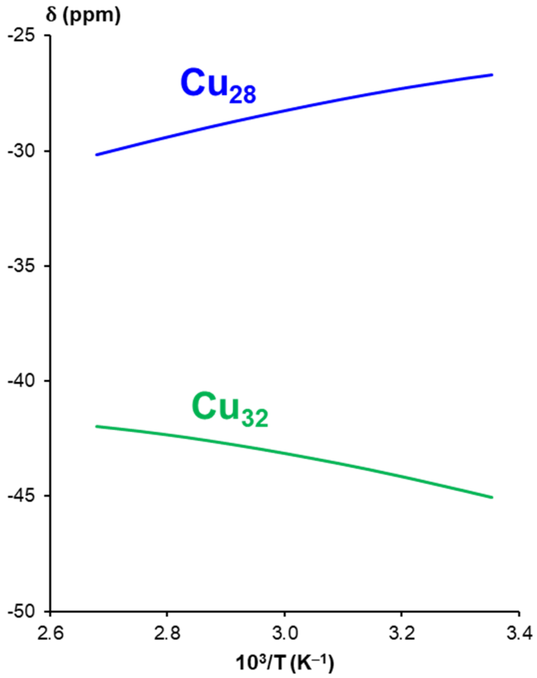

Figure S8. Curie plots for the ^{19}F atoms in the **$Cu_{28}SiF_6$** and **$Cu_{32}SiF_6$** nanojars in DMSO-d_6, illustrating the influence of the size of the nanojar on chemical shift.

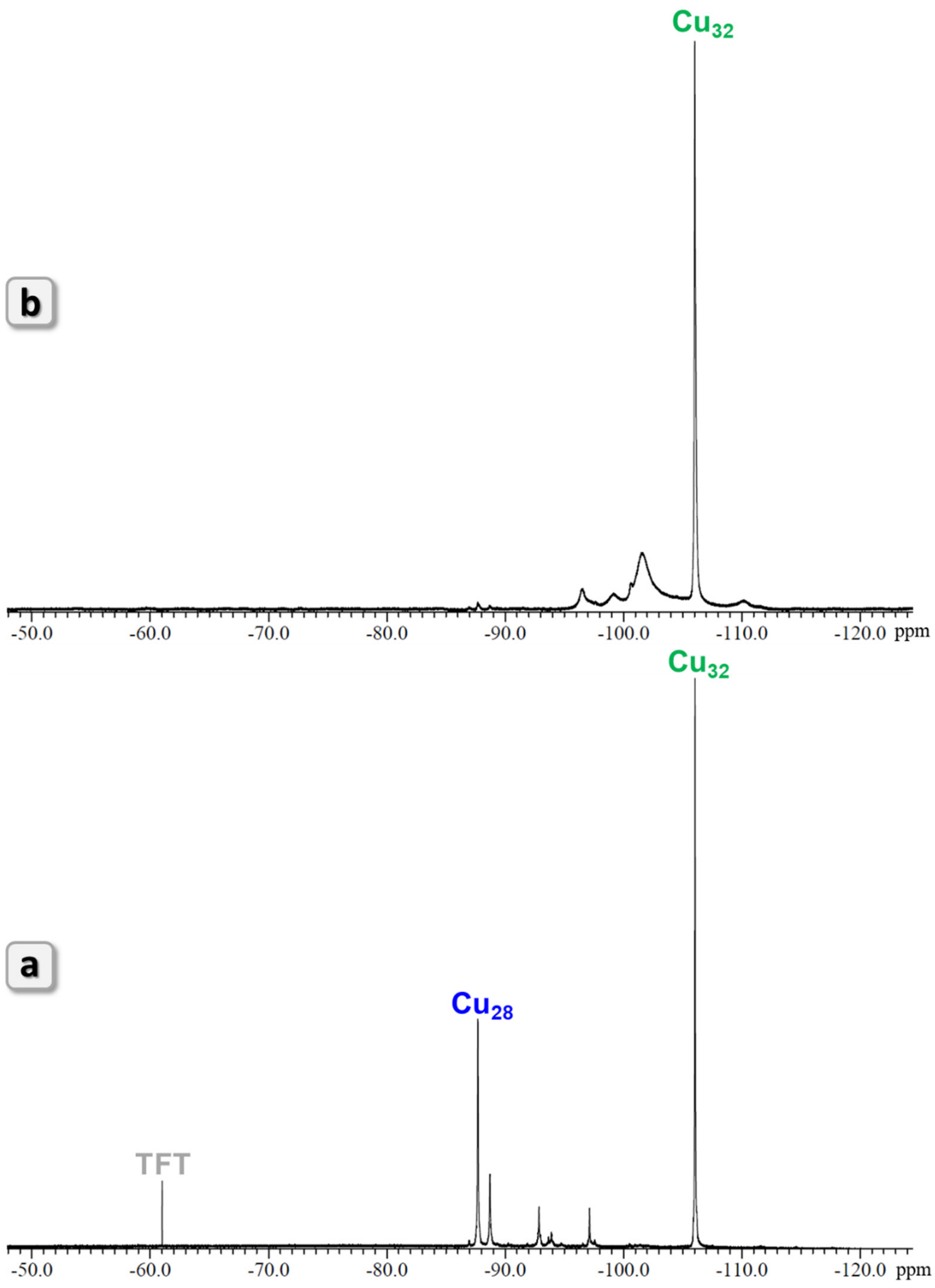

Figure S9. ^{19}F NMR spectra in DMSO-d_6 at ambient temperature of the $(Bu_4N)_2[SiF_6\subset\{Cu(OH)(pz)\}_n]$ (n = 28, 30, 32, 34) nanojar mixture a) before heating, and b) after the ^{1}H VT-NMR experiment (heating up to 150 °C). The $C_6H_5CF_3$ (TFT; b.p. 102 °C) internal reference was not included in the sample used for the ^{1}H VT-NMR experiment. Hence, for comparison purposes, the chemical shift of TFT in the spectrum before heating was not set to δ = 0.00 ppm.
